# Design and Evaluation of a *Inonotus obliquus*–AgNP–Maltodextrin Delivery System: Antioxidant, Antimicrobial, Acetylcholinesterase Inhibitory and Cytotoxic Potential

**DOI:** 10.3390/polym17152163

**Published:** 2025-08-07

**Authors:** Ana-Maria Stanoiu, Cornelia Bejenaru, Adina-Elena Segneanu, Gabriela Vlase, Ionela Amalia Bradu, Titus Vlase, George Dan Mogoşanu, Maria Viorica Ciocîlteu, Andrei Biţă, Roxana Kostici, Dumitru-Daniel Herea, Ludovic Everard Bejenaru

**Affiliations:** 1 Department of Surgery, Faculty of Medicine, Victor Babeş University of Medicine and Pharmacy Timişoara, 2 Eftimie Murgu Square, 300041 Timişoara, Romania; ana-maria.stanoiu@umft.ro; 2 Drug Research Center, Faculty of Pharmacy, University of Medicine and Pharmacy of Craiova, 2 Petru Rareş Street, 200349 Craiova, Romania; cornelia.bejenaru@umfcv.ro (C.B.); george.mogosanu@umfcv.ro (G.D.M.); maria.ciocilteu@umfcv.ro (M.V.C.); andreibita@gmail.com (A.B.); roxana.kostici@umfcv.ro (R.K.); ludovic.bejenaru@umfcv.ro (L.E.B.); 3 Department of Pharmaceutical Botany, Faculty of Pharmacy, University of Medicine and Pharmacy of Craiova, 2 Petru Rareş Street, 200349 Craiova, Romania; 4 Institute for Advanced Environmental Research, West University of Timişoara (ICAM–WUT), 4 Oituz Street, 300086 Timişoara, Romania; gabriela.vlase@e-uvt.ro (G.V.); ionela.bradu@e-uvt.ro (I.A.B.); titus.vlase@e-uvt.ro (T.V.); 5 Research Center for Thermal Analyzes in Environmental Problems, West University of Timişoara, 16 Johann Heinrich Pestalozzi Street, 300115 Timişoara, Romania; 6 Department of Pharmacognosy & Phytotherapy, Faculty of Pharmacy, University of Medicine and Pharmacy of Craiova, 2 Petru Rareş Street, 200349 Craiova, Romania; 7 Department of Instrumental and Analytical Chemistry, Faculty of Pharmacy, University of Medicine and Pharmacy of Craiova, 2 Petru Rareş Street, 200349 Craiova, Romania; 8 Department of Toxicology, Faculty of Pharmacy, University of Medicine and Pharmacy of Craiova, 2 Petru Rareş Street, 200349 Craiova, Romania; 9 National Institute of Research and Development for Technical Physics, 47 Dimitrie Mangeron Avenue, 700050 Iaşi, Romania; dherea@phys-iasi.ro

**Keywords:** *Inonotus obliquus*, silver nanoparticles, micro-spray encapsulation, antioxidant potential, antimicrobial screening, anti-acetylcholinesterase activity, in vitro cytotoxicity

## Abstract

*Inonotus obliquus*, a medicinal mushroom valued for its bioactive compounds, has not been previously characterized from Romanian sources. This study presents the first comprehensive chemical and biological screening of *I. obliquus*, introducing novel polymer-based encapsulation systems to enhance the stability and bioavailability of its bioactive constituents. Two distinct delivery systems were designed to enhance the functionality of *I. obliquus* extracts: (i) microencapsulation in maltodextrin (MIO) and (ii) a sequential approach involving preparation of silver nanoparticle-loaded *I. obliquus* (IO–AgNPs), followed by microencapsulation to yield the hybrid MIO–AgNP system. Comprehensive metabolite profiling using GC–MS and ESI–QTOF–MS revealed 142 bioactive constituents, including terpenoids, flavonoids, phenolic acids, amino acids, coumarins, styrylpyrones, fatty acids, and phytosterols. Structural integrity and successful encapsulation were confirmed by XRD, FTIR, and SEM analyses. Both IO–AgNPs and MIO–AgNPs demonstrated potent antioxidant activity, significant acetylcholinesterase inhibition, and robust antimicrobial effects against *Staphylococcus aureus*, *Bacillus cereus*, *Pseudomonas aeruginosa*, and *Escherichia coli*. Cytotoxicity assays revealed pronounced activity against MCF-7, HCT116, and HeLa cell lines, with MIO–AgNPs exhibiting superior efficacy. The synergistic integration of maltodextrin and AgNPs enhanced compound stability and bioactivity. As the first report on Romanian *I. obliquus*, this study highlights its therapeutic potential and establishes polymer-based nanoencapsulation as an effective strategy for optimizing its applications in combating microbial resistance and cancer.

## 1. Introduction

*Inonotus obliquus* (Ach. ex Pers.) Pilát, commonly known as Chaga mushroom, is a slow-growing medicinal fungus of the *Hymenochaetaceae* family [1,2,3,4,5,6]. For centuries, it has been utilized in traditional medicine across Russia, the Baltic countries, Eastern Europe, and parts of Asia to treat various ailments, including gastrointestinal disorders, inflammatory conditions, diabetes mellitus, and cancer [1,2,3,4,5,6]. Its therapeutic potential is attributed to a diverse array of bioactive compounds, including polysaccharides (notably β-glucans), triterpenoids, polyphenols (such as phenolic acids), and melanin-like pigments, which exhibit antioxidant, immunomodulatory, antitumor, and antidiabetic properties [1,2,3,4,5,6,7,8,9,10,11,12,13,14,15].

This parasitic fungus primarily colonizes birch trees (*Betula pendula*, *B. pubescens*) and, less commonly, other deciduous species such as *Fagus sylvatica* (European beech), forming a characteristic sterile conk or sclerotium [1,2,3,4,5,6,7]. The sclerotium appears as a black, cracked, charcoal-like mass enclosing a reddish-brown interior of obliquely aligned hyphal tubes [1,2,3,4,5,6,7,8]. These sclerotia, which persist on host trees for years, are traditionally harvested for medicinal use [1,2,3,4,5,6,7]. In Romania, wild *I. obliquus* is found in mountainous forested regions and is sporadically employed in folk remedies and artisanal health products [16]. However, despite its ethnomedicinal significance, Romanian *I. obliquus* remains understudied regarding its chemical composition and biological activity, representing a significant gap in the scientific literature.

Globally, the rising demand for functional foods and nutraceuticals has spurred renewed interest in *I. obliquus* [1,2,3,4,5,6,7,13,17]. Chaga-based products, including teas, extracts, powders, and capsules, are increasingly marketed for their health-promoting effects [1,2,3,4,5,6,7].

However, the chemical profile and corresponding bioactivity of natural compounds are profoundly influenced by a complex interplay of factors, including geographic origin, host tree species, environmental and climatic conditions, as well as extraction-related parameters such as solvent polarity, drying method, temperature, and pH [7,8,9,10,11,17,18,19,20,21,22,23,24,25,26,27,28,29,30,31].

Region-specific studies on Romanian botanical and fungal sources have provided robust, experimentally validated evidence that environmental conditions, geographic origin, host species, and extraction methodologies significantly influence the phytochemical composition and biological efficacy of natural extracts [22,23,24,25,26,27,28,29].

These investigations collectively demonstrate that variations in these factors generate distinct bioactive profiles, underscoring the necessity for careful consideration to ensure reproducibility and therapeutic reliability in natural product research [22,23,24,25,26,27,28,29].

Consequently, there is a critical need for localized studies to elucidate the unique phytochemical signatures of *I. obliquus* from Romanian ecosystems, which may differ substantially from those reported in other regions [1,2,3,4,5,6,7,8,9,10,11,12,13,14,16,17,18,19,20]. Given the well-documented influence of environmental and methodological factors on chemical composition and biological activity, the absence of comprehensive region-specific data represents a critical unmet need within the current research landscape [22,23,24,25,26,27,28,29].

Addressing this deficiency is essential, as local fungal populations are likely to exhibit distinct phytochemical profiles and bioactivities, necessitating targeted investigations to support their evidence-based and standardized application in health-related fields.

Concurrently, modern biomedical research faces two pressing global challenges: escalating antimicrobial resistance (AMR) and the rising prevalence of neurodegenerative diseases [31,32,33,34,35,36]. These issues necessitate novel therapeutic agents with innovative mechanisms of action [31,32,33,34,35,36,37,38,39]. Natural products, particularly those derived from fungi and medicinal plants, are actively explored for their dual antimicrobial and acetylcholinesterase (AChE) inhibitory activities due to their structural diversity, multi-target pharmacological profiles, and generally favorable safety profiles [1,2,4,5,6,7,36,37,38,39,40,41,42,43,44].

Despite its pharmacological promise, the practical application of *I. obliquus* is limited by several challenges [1,2,3,4,5,6,7,8,9,10,12,14,17,18,20,21]. Various secondary metabolites often exhibit poor water solubility, structural complexity, and limited stability, reducing bioavailability and clinical efficacy [23,28,29]. Additionally, the bitter taste and chitin-rich texture of raw Chaga hinder its use in direct consumption or food formulations [1,2,3,4,5,6,7,17]. To address these limitations, recent efforts have focused on nanotechnology and encapsulation strategies to enhance solubility, stability, palatability, and controlled delivery of Chaga-derived bioactive compounds [45].

Among nanocarrier systems, silver nanoparticles (AgNPs) have garnered significant attention due to their unique physicochemical and biomedical properties, including surface plasmon resonance, tunable morphology, and potent antimicrobial, antioxidant, and cytotoxic effects [29,31,33,34,46,47,48]. Functionalizing AgNPs with natural bioactive compounds can improve their pharmacokinetic profiles, enable targeted delivery, and promote synergistic therapeutic effects [31,33,34,37,39,46,49]. However, the development of hybrid systems combining *I. obliquus* with AgNPs, particularly using Romanian wild strains, remains largely unexplored.

Encapsulation technologies, particularly those employing food-grade carriers such as maltodextrin, offer a promising approach to overcome the formulation challenges of Chaga-based products [50,51]. Spray drying, in particular, facilitates the production of stable powders with improved solubility, taste masking, protection of thermolabile compounds, and controlled release capabilities [50,51,52]. Despite these advantages, spray drying encapsulation of *I. obliquus* has not been reported, representing a novel opportunity for product development.

In response to these challenges and research gaps, this study introduces several key innovations with scientific and practical relevance. It presents the first comprehensive mycochemical profiling of low-molecular-weight metabolites from wild-harvested Romanian *I. obliquus*, providing critical insight into its unique regional phytochemical fingerprint. Building on this chemical foundation, two novel delivery systems were developed to enhance the functional usability and application potential of *I. obliquus*. The first system involves spray drying encapsulation of powdered *I. obliquus* in a maltodextrin matrix to improve solubility, stability, and functional performance. The second system comprises a ternary formulation combining *I. obliquus* with AgNPs, subsequently encapsulated in a maltodextrin matrix via spray drying, to promote synergistic bioactivity and enhance delivery efficiency. Additionally, the biological efficacy of Romanian *I. obliquus* and the resulting Chaga-based hybrid systems, both before and after encapsulation, was evaluated through comprehensive physicochemical characterization and in vitro assays of antioxidant, antimicrobial, AChE inhibitory, and cytotoxic activities.

## 2. Materials and Methods

### 2.1. Chemicals and Reagents

All chemicals and reagents utilized in this study were of analytical grade to ensure experimental accuracy, reproducibility, and consistency across assays. Key reagents, including 2,2-diphenyl-1-picrylhydrazyl (DPPH), Folin–Ciocalteu reagent, dimethyl sulfoxide (DMSO), potassium persulfate, sodium acetate, sodium carbonate, sodium phosphate, ammonium molybdate, potassium chloride, 2,4,6-*tris*(2-pyridyl)-1,3,5-triazine (TPTZ), ferric chloride (FeCl_3_), ferrous sulfate heptahydrate (FeSO_4_·7H_2_O), and hydrochloric acid (HCl) were procured from Sigma-Aldrich (München, Germany). The 3-(4,5-dimethylthiazol-2-yl)-2,5-diphenyltetrazolium bromide (MTT) assay kit was sourced from AAT Bioquest (Pleasanton, CA, USA). Maltodextrin (dextrose equivalent: 16.5–19.5) was obtained from Carbosynth (Berkshire, UK). Additional reagents included AChE from *Electrophorus electricus*, 1-naphthyl acetate, Fast Blue B salt, Tris-HCl buffer solution (0.05 M, pH 7.8), and rivastigmine (used as a positive control), all procured from Sigma-Aldrich. The solvents used in this study included methanol, ethanol, and chloroform (Merck, Darmstadt, Germany). Ultrapure water, produced using a Milli-Q system (Merck, Darmstadt, Germany), was employed throughout all experimental procedures.

### 2.2. Fungal Material (IO Sample)

The wild-grown *I. obliquus* was harvested in November 2023 from a distinct ecological area in central-western part of Romania: Râu de Mori Commune, on the shore of the Gura Apelor Lake, Retezat Mountains, Hunedoara County, Transylvania Region. The fungal material (*Inonotus obliquus*) was authenticated and deposited in the Herbarium of the Department of Pharmaceutical Botany, Faculty of Pharmacy, University of Medicine and Pharmacy of Craiova, Romania (voucher code: INON-OBQ-2023-0811). After air-drying for 24 h in brown paper bags under controlled conditions (room temperature, cool and dark environment), the material was prepared for extraction and analysis. The study did not involve any endangered or protected plant or fungal species.

### 2.3. Preparation of AgNPs

AgNPs (20–40 nm) were synthesized following previously validated protocols [37,39,49].

### 2.4. Cell Lines

Human cancer cell lines, MCF-7 (breast), HCT116 (colorectal), and HeLa (cervical), were sourced from ATCC (Manassas, VA, USA). Cells were maintained in DMEM (Gibco, UK) supplemented with 10% FBS and 1% antibiotic–antimycotic (Sigma-Aldrich, Germany), under standard culture conditions (37 °C, 5% CO_2_, humidified atmosphere) [39,53,54].

### 2.5. Bacterial Strains

Bacterial strains, including *Staphylococcus aureus* (ATCC 29213), *Bacillus cereus* (ATCC 14579), *Pseudomonas aeruginosa* (ATCC 27853), and *Escherichia coli* (ATCC 25922), were sourced from ATCC (Manassas, VA, USA). Strains were maintained and cultured according to ATCC guidelines to ensure viability and consistency [39,53,54].

### 2.6. Fungal Sample Preparation for Mycochemical Screening

Air-dried *I. obliquus* samples were ground using a Fritsch Pulverisette planetary mill (Idar-Oberstein, Germany) at 800 rpm for 15 min under controlled conditions (22 °C). The powder was then sieved (ASTM standard) to isolate particles sized 0.15–0.20 mm. Extraction was performed by sonication (Elmasonic, Singen, Germany) at 40 °C and 70 Hz for 45 min using 30 mL methanol as solvent. All extractions were carried out in triplicate to ensure reproducibility.

### 2.7. GC–MS Analysis

Gas chromatography–mass spectrometry (GC–MS) analysis was performed on a Shimadzu GCMS-QP2020 NX system equipped with a ZB-5MS capillary column (50 m × 0.20 mm, 0.33 μm film thickness; Agilent Technologies, Santa Clara, CA, USA). Helium served as the carrier gas at 1 mL/min. The oven temperature program started at 50 °C (2 min hold), ramped at 3 °C/min to 300 °C, and held for 2 min. Injector and interface temperatures were set at 280 °C and 225 °C, respectively. Ionization was conducted at 80 eV with a 1 min solvent delay. The mass spectrometer source and quadrupole temperatures were maintained at 230 °C and 140 °C, respectively. Analyses were conducted in triplicate.

Compound identification was achieved by matching mass spectra against the NIST 2.0 database (NIST, Gaithersburg, MD, USA) and supported by literature comparison. Retention indices were calculated using the Van den Dool and Kratz method with a C7–C40 n-alkane standard, while Kováts retention indices were obtained via logarithmic interpolation to improve identification accuracy [53,54,55,56].

### 2.8. MS Analysis

Mass spectrometry analysis was conducted using an electrospray ionization–quadrupole time-of-flight system (ESI–QTOF–MS; Bruker Daltonics, Bremen, Germany) operating in positive ion mode over a mass range of 50–3000 *m*/*z* with a scan rate of 2.0 scans/s. Collision energies varied between 20 and 80 eV, and the source block temperature was set at 80 °C. Biomolecule identification was performed by matching spectra against the NIST/NBS-3 library and supported by relevant literature [53,54].

### 2.9. Spray Drying Process

Spray drying was carried out using a Mini Spray Dryer B-290 (Büchi, Flawil, Switzerland) under optimized conditions: feed flow rate of 8 mL/min, inlet temperature of 125 °C, outlet temperature of 70 °C, airflow of 30 m^3^/h, compressor pressure of 0.05 MPa, and a 0.7 mm nozzle diameter. The process was conducted at approximately 80% relative humidity with 100% suction airflow [53,54,57,58].

### 2.10. Preparation of I. obliquus–AgNP (IO–AgNP) System

The IO–AgNP system was prepared by mixing *I. obliquus* powder (obtained as previously described) with an AgNP solution at a 1:2 mass ratio. This ratio was selected based on our prior studies to ensure an optimal balance between nanoparticle stability and the efficient incorporation of bioactive compounds [37,38,39,49,59,60]. The mixture was subjected to ultrasonic treatment at 35 °C for 40 min to promote uniform dispersion and interaction between the components. The resulting suspension was then filtered using 185 mm filter paper and dried in an oven at 45 °C for 8 h. All experiments were conducted in triplicate to ensure reproducibility.

### 2.11. Preparation of Maltodextrin–I. obliquus (MIO) System

The MIO system was prepared by dissolving 2.2 g each of dried *I. obliquus* and maltodextrin in 50 mL ultrapure water, followed by thorough homogenization to ensure uniform dispersion and effective encapsulation, as validated in similar systems [53,54]. The mixture was incubated at 30 °C with continuous stirring for 30 min to promote interaction between bioactive compounds and the carrier matrix. After incubation, the suspension was centrifuged for 8 min and filtered through a 0.45 μm Whatman membrane. The clear filtrate was spray-dried, and the resulting powder was stored in opaque, airtight containers at 25 °C to maintain stability. All experiments were performed in triplicate to ensure reproducibility.

### 2.12. Preparation of Maltodextrin–IO–AgNP (MIO–AgNP) System

The MIO–AgNP system was prepared by mixing the IO–AgNPs formulation with maltodextrin at a 1:1 mass ratio, following the same procedure established for the MIO system [53,54]. Experiments were conducted in triplicate to ensure reproducibility.

### 2.13. Characterization of New Prepared I. obliquus-Based Systems

#### 2.13.1. FTIR Spectroscopy

FTIR spectra were recorded using a Shimadzu AIM-9000 spectrometer equipped with ATR (Shimadzu, Tokyo, Japan). Measurements were performed over 20 scans with a 4 cm^−1^ resolution across the 4000–400 cm^−1^ range. Peak assignments were interpreted based on established literature.

#### 2.13.2. XRD Analysis

X-ray diffraction (XRD) patterns were acquired using a Bruker AXS D8 Advance diffractometer (Karlsruhe, Germany) with CuKα radiation (λ = 0.1541 nm). Measurements were performed over a 2θ range of 5° to 80°, with a 0.02° step size and 2 s per step scan rate. The system included a rotating sample stage and temperature control units for low and high temperatures. Data analysis was conducted using DIFFRAC.EVA software version 7.0 and compared to reference patterns from the ICDD Powder Diffraction Database (ICDD file 04-015-9120). Crystallite size, lattice parameters, and phase composition were determined using the whole powder pattern fitting (WPPF) method via TOPAS software version 7.1.0 for accurate structural characterization.

#### 2.13.3. Scanning Electron Microscopy (SEM) Analysis

Surface morphology and elemental composition were examined using a JEOL JSM-IT200 InTouchScope™ SEM (Freising, Germany) with a field emission gun (FEG) and energy-dispersive X-ray spectroscopy (EDS). Samples were mounted on carbon tape and sputter-coated with a 10 nm gold layer for conductivity enhancement. Imaging was conducted at 15 kV accelerating voltage and a 10 mm working distance, with magnifications ranging from 100× to 10,000×. EDS analysis was conducted to quantify elemental distributions, with spectra collected at multiple regions to ensure representativeness.

#### 2.13.4. DLS Analysis

For dynamic light scattering (DLS) analysis, particle size distribution (PSD) was measured using a Microtrac Nanotrac Wave II (Microtrac Retsch GmbH, Montgomeryville, PA, USA). Samples were dispersed in deionized water and analyzed at 23 °C with a laser wavelength of 780 nm and a scattering angle of 180°. Each measurement was performed in triplicate, with results reported as the mean hydrodynamic diameter and polydispersity index (PDI).

#### 2.13.5. Encapsulation Efficiency, Loading Capacity, and Yield

The encapsulation efficiency (EE%), loading capacity (LC%), and encapsulation yield (EY%) of *I. obliquus* and the IO–AgNP system were determined using Equations (1), (2), and (3), respectively [49,53,54,57,58]:

(1) $$\mathrm{EE}\;(\%)\;=\;\frac{mass\;of\;encapsulated\;compound\;(g)\;}{mass\;of\;raw\;material\;(g)}\times 100$$

(2) $$\mathrm{LC}\;(\%)=\frac{mass\;of\;capsules\;(g)}{mass\;of\;raw\;material\;\left(g\right)+mass\;of\;maltodextrin\;(g)}\times 100$$

(3) $$\mathrm{EY}\;(\%)=\frac{mass\;of\;capsules\;\left(g\right)-mass\;of\;raw\;material\;(g)}{mass\;of\;raw\;materials\;(g)}\times 100$$

Quantification was carried out using a PerkinElmer Lambda 35 UV-Vis spectrophotometer (Waltham, MA, USA). Samples (20 mg) underwent ultrasound-assisted extraction (UAE) at 70 kHz in 25 mL of hydrochloric acid–ethanol–chloroform (3:2:2, *v*/*v*/*v*) for 45 min at 22 °C. Following extraction, samples were centrifuged at 6000 rpm for 10 min, and the supernatant was analyzed at 280 nm using an ethanol–chloroform (1:1, *v*/*v*) blank. All measurements were performed in triplicate using a 10 mm quartz cuvette [49,53,54,57,58].

### 2.14. Thermal Analysis

To evaluate the thermal stability and decomposition profiles of the samples, thermo-gravimetric analysis (TGA) and differential scanning calorimetry (DSC) were performed using a Mettler Toledo TG/DSC3+ thermal analyzer equipped with a high-temperature thermogravimetry/differential thermal analysis (TG/DTA) sensor. Samples were analyzed in 40 μL aluminum crucibles under a synthetic air atmosphere (purity 5.0), with a flow rate of 50 mL/min. The temperature range spanned 25–400 °C at a heating rate of 10 °C/min to ensure consistent thermal decomposition data. Calibration of the instrument was performed using indium and zinc standards to ensure accuracy and precision in temperature and enthalpy measurements.

### 2.15. Estimation of Total Phenolic Content and Antioxidant Activity

The total phenolic content (TPC) and antioxidant activity of *I. obliquus* (IO), IO–AgNP system, modified IO (MIO) system, and MIO–AgNP system were systematically evaluated using standardized biochemical assays (ferric reducing antioxidant power (FRAP) and DPPH radical scavenging assays). All experiments were conducted in triplicate to ensure reproducibility and statistical reliability.

#### 2.15.1. Sample Preparation Procedure

For extraction, 0.40 g of each sample (*I. obliquus*, IO–AgNPs, MIO, or MIO–AgNPs) was suspended in 10 mL of 70% (*v*/*v*) ethanol and agitated continuously at 23 °C for 10 h to maximize phenolic compound extraction. The mixtures were then centrifuged at 5000 rpm (approximately 3000× *g*) for 15 min to separate the supernatant from solid residues. The supernatant was collected and stored at 4 °C for subsequent TPC and antioxidant activity analyses. The same extraction protocol was applied to the IO–AgNP system, MIO system, and MIO–AgNP system to ensure methodological consistency.

#### 2.15.2. TPC Assay

Total phenolic content (TPC) was determined using the Folin–Ciocalteu assay, following established methods [49,53,54]. In brief, 100 μL of each extract was mixed with 500 μL of Folin–Ciocalteu reagent (1:10 dilution in deionized water) and 400 μL of 7.5% (*w*/*v*) sodium carbonate solution. After incubating at 40 °C for 30 min, absorbance was recorded at 765 nm using a FLUOstar Optima UV–Vis spectrometer (BMG Labtech, Offenburg, Germany). TPC values were calculated from a gallic acid calibration curve (R^2^ = 0.9637) using the linear Equation (4):

*y* = 0.0022*x* + 0.0225 (4)

Results were expressed as milligrams of gallic acid equivalents (GAE) per gram of sample (mg GAE/g).

#### 2.15.3. FRAP Assay

The FRAP assay was performed to assess the reducing capacity of the samples, following previously described protocols [49,53,54,59,60]. Sample extracts (50 μL) were mixed with 1.5 mL of FRAP reagent (containing 10 mM TPTZ, 20 mM FeCl_3_, and 300 mM acetate buffer, pH 3.6) and incubated at 37 °C for 4 min. Absorbance was measured at 595 nm using a FLUOstar Optima UV–Vis spectrometer. Antioxidant activity was quantified as millimolar Fe^2+^ equivalents (mM Fe^2+^) using Equation (5):

(5) $$\mathrm{FRAP}\;=\;\frac{C_{{Fe}^{2+}\;}\times \;F}{V}$$

where *C_Fe_*^2+^ is the Fe^2+^ concentration (nM) derived from the calibration curve (*R*^2^ = 0.9997; *y* = 0.0016*x* + 0.0915), *F* is the dilution factor, and *V* is the sample volume (μL).

#### 2.15.4. DPPH Radical Scavenging Assay

The DPPH radical scavenging activity was determined according to previous studies [53,54,55,56]. Sample extracts (100 μL) were mixed with 3.9 mL of 0.1 mM DPPH solution in methanol and incubated in the dark at 23 °C for 30 min. Absorbance was measured at 520 nm using a FLUOstar Optima UV–Vis spectrometer. The percentage of DPPH inhibition was calculated using Equation (6):

(6) $$\mathrm{Inh}\;(\%)\;=\frac{\left(A_{0\;\;}-\;A_{1}\right)}{A_{0}}\times 100$$

where *A*_0_ is the absorbance of the control (DPPH solution without sample) and *A*_1_ is the absorbance of the sample. The half-maximal inhibitory concentration (IC_50_, μg/mL) was determined from the inhibition percentage plotted against sample concentrations.

### 2.16. AChE Inhibitory Assay

The neuroprotective potential of the previously prepared samples (*I. obliquus*, IO–AgNPs, MIO, and MIO–AgNPs) was assessed using a microplate-based AChE inhibitory assay. Serial dilutions of each sample were prepared to establish a concentration gradient. The enzymatic reaction was initiated by the addition of 1-naphthyl acetate (3 mg/mL in ethanol) as the substrate, followed by AChE enzyme (3.33 U/mL). Fast Blue B salt (3 mg/mL in water) was used as a chromogenic reagent to detect enzymatic activity. Absorbance was measured at 595 nm using a microplate reader. Rivastigmine served as the positive control [42,44].

### 2.17. Antimicrobial Activity

The antimicrobial activity of *I. obliquus*, IO–AgNPs, MIO, and MIO–AgNPs was evaluated against a panel of bacterial strains using agar well diffusion, minimum inhibitory concentration (MIC), and minimum bactericidal concentration (MBC) assays. All experiments were performed in triplicate to ensure reproducibility.

#### 2.17.1. Agar Well Diffusion Assay

The agar well diffusion assay was performed following established protocols [54,55]. Müller–Hinton agar plates were inoculated with microbial suspensions standardized to 0.5 McFarland (1.5 × 10^8^ CFU/mL). Wells (6 mm diameter) were filled with 50 μL of sample solutions at concentrations ranging from 100 to 200 μg/mL in 25% DMSO [39,53,54,59]. Plates were incubated at 37 °C for 24 h, and inhibition zone diameters were measured using a digital caliper.

#### 2.17.2. MIC and MBC Determination

Minimum inhibitory concentration (MIC) and minimum bactericidal concentration (MBC) values were measured using the microbroth dilution method in Müller–Hinton broth. Serial dilutions of samples (100–200 μg/mL) were prepared in 96-well plates, followed by the addition of 100 μL microbial suspension standardized to 0.5 McFarland. After 24 h of incubation at 37 °C, MIC was defined as the lowest concentration preventing visible growth, assessed by optical density at 600 nm using a T90+ UV–Vis spectrophotometer (PG Instruments, Wibtoft, UK). MBC was determined by sub-culturing 10 μL from wells without growth onto Müller–Hinton agar plates, incubated for an additional 14 h at 37 °C. [39,53,54,59]. The MBC corresponded to the lowest concentration showing no bacterial growth.

### 2.18. Cell Culture and Cytotoxicity Assessment

#### 2.18.1. Cell Culture and Treatment

The cytotoxicity of *I. obliquus*, IO–AgNPs, MIO, and MIO–AgNPs was evaluated using MCF-7 (breast cancer), HCT116 (colorectal cancer), and HeLa (cervical cancer) cell lines (ATCC, Manassas, VA, USA). Cells were cultured in Dulbecco’s Modified Eagle Medium (DMEM) supplemented with 10% FBS and 1% antibiotic–antimycotic solution, maintained at 37 °C in a humidified 5% CO_2_ atmosphere. Cells were seeded at 4 × 10^3^ cells/well in 96-well plates and incubated for 24 h to achieve 90% confluency. The medium was replaced with fresh DMEM containing sample concentrations of 75, 100, 125, 150, 175, and 200 μg/mL [39,53,54,59,60]. Positive (untreated cells) and negative (non-viable cells) controls were included. All conditions were tested in triplicate.

#### 2.18.2. Cell Viability Assessment

Cell viability was determined using the MTT assay. After 24 h of treatment, the culture medium was removed, and 25 μL of MTT solution (5 mg/mL) was added to each well. Plates were incubated for 2 h at 37 °C to allow formazan formation, which was subsequently dissolved by adding 100 μL of DMSO. Absorbance was read at 540 nm using a Synergy HTX Multi-Mode Microplate Reader (Agilent Technologies, USA). Cell viability (%) was calculated according to Equation (7).

(7) $$\mathrm{CV}\;(\%)=\frac{\left({OD}_{sample}-{OD}_{blank}\right)}{\left({OD}_{control}-{OD}_{blank}\right)}\times 100$$

where *OD_sample_* is the optical density of treated cells, *OD_control_* is the optical density of untreated cells, and *OD_blank_* is the optical density of the culture medium. IC_50_ values (concentration reducing cell viability by 50%) were determined by plotting cell viability against sample concentrations (75, 100, 125, 150, 175, and 200 μg/mL) [39,53,54,59,60,61].

### 2.19. Statistical Analysis

Experiments were conducted in triplicate, and data are presented as mean ± standard deviation (SD). Statistical significance was assessed using Student’s *t*-test for two-group comparisons and one-way ANOVA followed by Tukey’s HSD post hoc test for multiple comparisons. Analyses were performed with Microsoft Excel 2019 (Microsoft Corporation, Redmond, WA, USA). Differences with *p*-values less than 0.05 were considered statistically significant.

## 3. Results

### 3.1. Mycochemical Screening

The chemical complexity of Romanian *I. obliquus* was investigated using a dual-platform analytical approach that combined GC–MS and ESI–QTOF–MS. This integrated methodology enabled comprehensive profiling of both volatile and semi-volatile low-polarity compounds as well as polar, thermolabile metabolites, providing a detailed phytochemical map of *I. obliquus* from this specific geographical region.

#### 3.1.1. GC–MS Analysis

*I. obliquus* is known to contain a wide array of non-polar bioactive compounds, such as lanostane-type triterpenoids, sterols, and hydrocarbons, which require thermal desorption and electron ionization for effective separation and identification [1,2,3,4,5,6,7,8]. GC–MS offers high chromatographic resolution and delivers retention indices alongside characteristic fragmentation patterns, which are essential for the reliable structural confirmation of these compounds. Additionally, GC–MS enables semi-quantitative assessment of compound abundance through relative peak area integration, offering valuable insights into the dominant chemical constituents. Importantly, many GC–MS-detected compounds contain functional groups, hydroxyl, ketone, ester, and carboxyl, that can interact with AgNPs, affecting surface functionalization, stability, and controlled release profiles [62,63,64]. Therefore, GC–MS analysis was not only crucial for characterizing the chemical diversity of *I. obliquus* but also for pinpointing molecular candidates relevant to the rational design of AgNP-based engineered delivery systems (IO–AgNPs and MIO–AgNPs).

**Figure 1:**
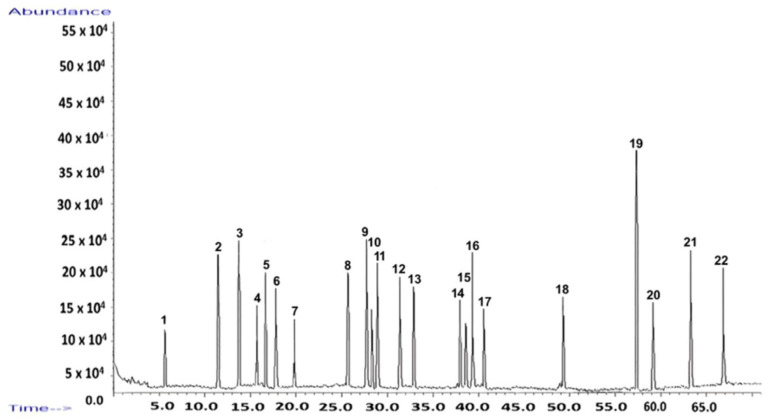
Total ion chromatogram of *I. obliquus* sample.

The chromatographic fingerprint (Figure 1) visually illustrates the complexity of the extract, with each peak corresponding to a specific compound detailed in Table 1.

GC–MS analysis identified 22 distinct compounds in the *I. obliquus* extract, collectively accounting for 59.70% of the total ion chromatogram (Figure 1; Table 1). These compounds belong to several key chemical classes:

- Triterpenoids and sterols: inotodiol (14.42%), lupeol (2.55%), lupenone (2.61%), β-sitosterol (2.33%), ergosterol (1.91%), trametenolic acid (2.14%);
- Fatty acid esters: methyl palmitate (1.69%), methyl linoleate (2.45%);
- Sesquiterpenes and aromatic compounds: α-curcumene (1.73%), α-turmerone (2.71%), coumarin (1.94%), benzyl benzoate (1.81%);
- Hydrocarbons: hexadecane, heptadecane, octadecane;
- Other notable compounds: betulin (2.29%), brassicasterol (1.96%).

Among these, inotodiol was the most abundant compound, reinforcing its prominence in the chemical composition of *I. obliquus*. The GC–MS fingerprint (Figure 1) demonstrates the extract’s chemical diversity and richness in bioactive molecules.

#### 3.1.2. ESI–MS Analysis

The selection of ESI–MS was driven by the necessity for in-depth molecular characterization to facilitate the development of AgNP-based carrier systems. This technique enables the precise identification of key bioactive phytoconstituents, providing crucial in-sights into their chemical stability, potential interactions with AgNPs, and relevance to controlled-release mechanisms.

**Figure 2:**
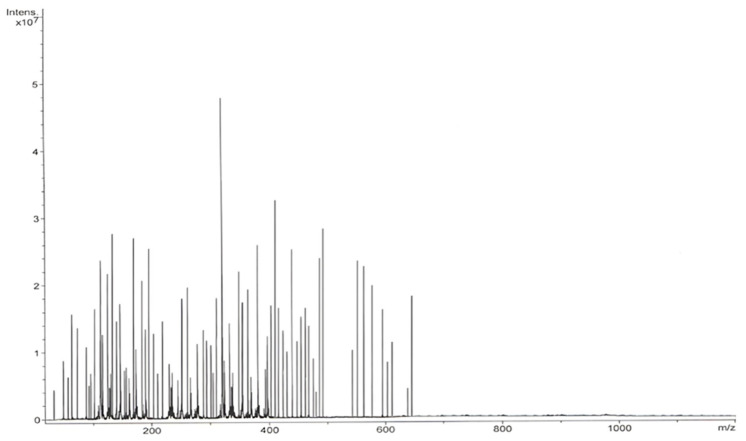
The mass spectrum of *I. obliquus* sample.

ESI–MS analysis of *I. obliquus* revealed a diverse array of bioactive compounds, including amino acids, coumarins, terpenes, fatty acids, flavonoids, phenolic acids, phytosterols, styrylpyrones, hydrocarbons, esters, and other polyphenols (Figure 2; Table 2).

These results are consistent with previous studies on *I. obliquus* from different geo-graphic regions, confirming the complex and rich phytochemical profile of the Romanian samples and highlighting their broad therapeutic potential.

#### 3.1.3. VOC Analysis

The sensory and therapeutic characteristics of *I. obliquus* are strongly influenced by its volatile organic compounds (VOCs), which contribute not only to its distinct aroma and flavor profile but also to its biological activities, including antimicrobial, anti-inflammatory, and immunomodulatory effects [1,2,3,4,5,6,7,8,9,72,73,74,75,76,77,78]. To evaluate the impact of AgNP incorporation and maltodextrin encapsulation on the integrity of these bioactive volatiles, a comprehensive VOC analysis was conducted.

**Figure 3:**
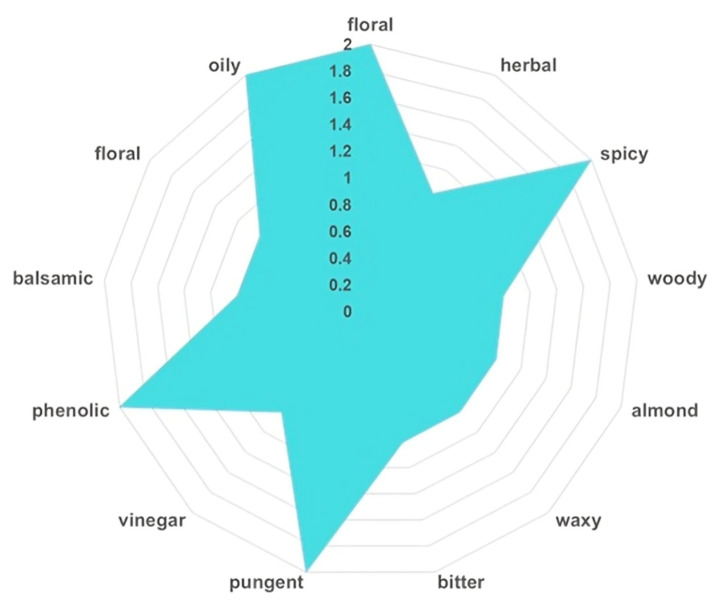
The VOC sensory profile of the constituents identified in the *I. obliquus* sample. VOC: Volatile organic compound.

The results are presented in Table 3 and Figure 3, highlighting the principal constituents that define the unique sensory complexity of native *I. obliquus*.

A total of 17 key VOCs were identified in the *I. obliquus* sample, each associated with specific odor descriptors (Table 3), collectively contributing to its multifaceted aroma profile.

### 3.2. Engineered Hybrid System

#### 3.2.1. FTIR Analysis

FTIR spectroscopy was employed to investigate the molecular interactions, such as chemical bonding and surface coordination, driving the formation and stabilization of the hybrid system (IO–AgNPs) composed of AgNPs and bioactive constituents from *I. obliquus*. This technique served a dual role: confirming the successful synthesis of the IO–AgNP composite and elucidating the role of maltodextrin in the encapsulation process leading to the formation of MIO and MIO–AgNP systems. The FTIR analyses offered critical insights into the molecular architecture and stabilization mechanisms underlying these multifunctional constructs.

**Figure 4:**
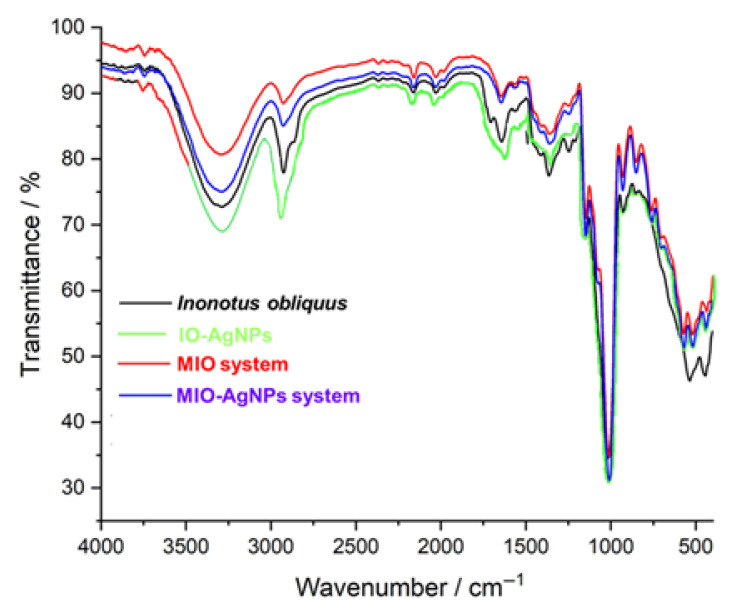
FTIR spectra of *I. obliquus* sample (black line), IO–AgNPs (green line), MIO (red line), and MIO–AgNPs (blue line) systems. FTIR: Fourier-transform infrared; IO–AgNPs: *I. obliquus*–silver nanoparticles; MIO: Maltodextrin—*I. obliquus*; MIO–AgNPs: Maltodextrin—*I. obliquus*–silver nanoparticles.

The FTIR spectrum of the *I. obliquus* sample (Figure 4, black line) exhibits a diverse range of absorption bands indicative of multiple biomolecular classes. Prominent peaks include a broad band at approximately 3291 cm^−1^, associated with O–H and N–H stretching vibrations from carbohydrates, polyphenols, and amino acids; a peak at 2928 cm^−1^, corresponding to C–H stretching of aliphatic groups in fatty acids and phytosterols; and bands at 1708 and 1636 cm^−1^, attributed to C=O stretching of phenolic acids, coumarins, and organic acids, as well as C=C stretching of styrylpyrones and flavonoids [5,8,19,20,21,37,38,39,52,53,54,59,60,79,80,81]. Additional peaks at 1368 cm^−1^ (C–H bending) and 534–444 cm^−1^ (out-of-plane bending of aromatic rings) suggest the presence of terpenes and phenolic compounds [5,8,19,20,21,37,38,39,52,53,54,59,60]. This spectrum highlights the complex chemical composition of the *I. obliquus* sample, encompassing amino acids, coumarins, styrylpyrones, terpenes, fatty acids, phenolic acids, flavonoids, phytosterols, organic acids, and carbohydrates.

The FTIR spectrum of the MIO system (Figure 4, red line) exhibits characteristic absorption bands reflecting contributions from both *I. obliquus* and maltodextrin components. A broad peak at 3296 cm^−1^ is attributed to O–H stretching vibrations from hydroxyl groups in polysaccharides of both *I. obliquus* and maltodextrin [5,8,53,54,57,58,78]. The band at 2928 cm^−1^ corresponds to C–H stretching of aliphatic chains, while the peak at 1643 cm^−1^ is assigned to C=O stretching vibrations from maltodextrin carbonyl groups and *I. obliquus*-derived phenolic compounds [53,54,57,58,78,79,80,81]. Additional bands include 1361 cm^−1^ (C–H bending), 1146 cm^−1^ (C–O–C stretching of glycosidic linkages), and 578–515 cm^−1^ (out-of-plane bending of aromatic rings) from *I. obliquus* metabolites [5,9,72,78,79,80,81]. Compared to the native *I. obliquus* spectrum, the MIO system displays increased intensity at 1643 cm^−1^ and 1146 cm^−1^, indicative of the enhanced maltodextrin matrix contribution [53,54,57,58]. The broadening of the 3296 cm^−1^ band suggests intensified hydrogen bonding interactions due to overlapping hydroxyl groups from both components [53,54,57,58]. These spectral modifications confirm successful encapsulation, preservation of molecular integrity, and the formation of a stable polysaccharide-rich matrix [53,54,57,58]. Furthermore, the observed intensity variations and band broadening imply molecular interactions between *I. obliquus* phytochemicals and maltodextrin, contributing to a well-integrated encapsulated system [53,54,57,58].

The FTIR spectrum of the IO–AgNP system (Figure 4, green line) showcases key vibrational features reflecting the *I. obliquus* matrix and its interaction with AgNPs [39,62,63,64]. A broad absorption band at 3300–3400 cm^−1^, attributed to O–H stretching of hydroxyl groups from polysaccharides and phenolic compounds, and a peak at 2900 cm^−1^, indicative of C–H stretching from aliphatic chains in fatty acids and sterols, highlight Chaga’s native composition. In the fingerprint region (1600–1000 cm^−1^), a C=C aromatic stretch at 1600 cm^−1^ and C–O, C–C, and C–N vibrations between 1400 and 1000 cm^−1^, linked to polysaccharides, phenolic compounds, proteins, and their bioactive ligands (e.g., hydroxyl and carbonyl groups from phenols, polysaccharide hydroxyls, and amine groups from proteins), are evident [1,2,3,4,5,9,62,63,64]. Subtle shifts and intensity changes, including a redshift and broadening of the 3300–3400 cm^−1^ band and a shift in the C=O band near 1700 cm^−1^, suggest interactions between citrate- and surfactant-coated AgNPs and these ligands [39,49]. Enhanced peak definition in the 1400–1000 cm^−1^ region, likely due to C–O or C–N coordination with silver (Ag), supports AgNP-induced hydrogen bonding alterations, confirming successful incorporation via mechanical alloying [39]. Furthermore, vibrational bands at approximately 1632, 1389, 1114, and 675 cm^−1^, potentially reflecting citrate-coated AgNPs stabilized by surfactant and *I. obliquus* ligands, shift to 1642, 1392, 1118, and 681 cm^−1^, indicating binding via O–H, C=O, N–H, and C–O groups [39,49]. These spectral changes validate the formation of a stable IO–AgNPs hybrid system, driven by robust AgNP–phytochemical interactions and structural reorganization of the bioactive matrix.

The FTIR spectrum of the MIO–AgNP system (Figure 4, blue line) displays distinct vibrational bands reflecting the IO–AgNP core and maltodextrin matrix [1,2,3,4,9,53,54,57,58,62,63,64]. A broad O–H stretching band at 3296 cm^−1^ indicates contributions from hydroxyl groups in *I. obliquus* polysaccharides, phenolic compounds, and maltodextrin, while a C–H stretching vibration at 2928 cm^−1^ corresponds to aliphatic chains from *I. obliquus* lipophilic components. In the fingerprint region, notable absorption bands appear at 1643 cm^−1^ (C=O stretching from maltodextrin carbonyls and Chaga phenolics), 1361 cm^−1^ (C–H bending), 1146 cm^−1^ (C–O–C stretching of maltodextrin glycosidic linkages), and 578–515 cm^−1^ (aromatic ring deformations from Chaga compounds) [1,2,3,4,9,53,54,57,58,62,63,64]. Compared to the unencapsulated IO–AgNP spectrum, the MIO–AgNP system exhibits a broadened and intensified O–H band at 3296 cm^−1^ and increased absorption at 1643 and 1146 cm^−1^, highlighting the dominant maltodextrin matrix contribution and enhanced hydrogen bonding [53,54,57,58]. The IO–AgNP spectrum’s characteristic AgNP–phytochemical interactions, such as the redshift of the C=O band from 1632 to 1642 cm^−1^ and sharpening in the 1400–1000 cm^−1^ region, are diminished in MIO-AgNPs, indicating that the maltodextrin coating shields and stabilizes the nanoparticle (NP) interface [39,53,54]. These spectral changes confirm successful encapsulation, yielding a structurally integrated hybrid system [53,54]. The modifications underscore maltodextrin’s protective role in maintaining the chemical integrity of bioactive compounds and modulating intermolecular interactions, suggesting enhanced stability for the newly prepared system.

#### 3.2.2. XRD Analysis

**Figure 5:**
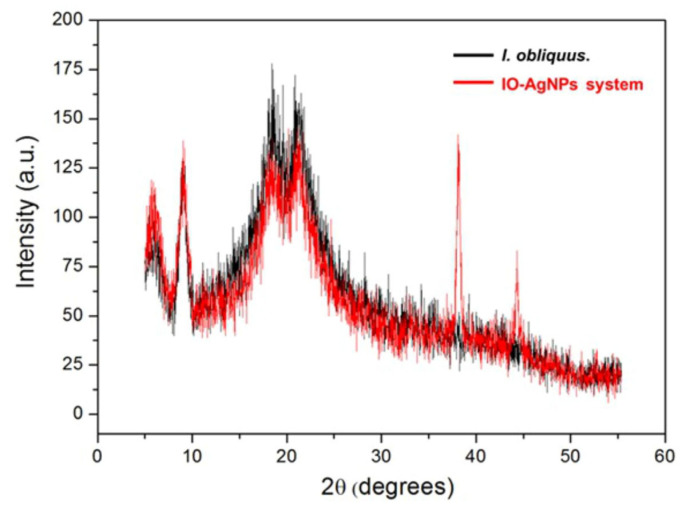
XRD patterns of the *I. obliquus* sample (black line) and the IO–AgNP system (red line). XRD: X-ray diffraction.

The XRD patterns provided in Figure 5 offer valuable insights into the structural characteristics and phase composition of the *I. obliquus* and IO–AgNP systems.

The black line, corresponding to the *I. obliquus* sample (Figure 5), exhibits a broad diffraction peak centered around 2θ (12.78° and 28.21°), which is indicative of an amorphous structure. This broad halo is consistent with the presence of a complex, heterogeneous matrix of biomolecules typically found in fungal biomass [82,83].

In contrast, the red line, corresponding to the IO–AgNP system (Figure 5), retains the broad amorphous peak of *I. obliquus* while exhibiting additional sharp, distinct diffraction peaks at approximately 2θ (27.81°, 38.15°, 64.4°, and 78.5°). These peaks are characteristic of the face-centered cubic (FCC) structure of metallic Ag, as referenced in Joint Committee on Powder Diffraction Standards (JCPDS) Card No. 04-0783 [37,39,49,63,64]. This alignment confirms the successful incorporation of crystalline AgNPs within the *I. obliquus* matrix. The coexistence of the amorphous halo with AgNP-specific peaks suggests that the NPs are embedded within the fungal matrix without causing significant disruption to its native amorphous structure, supporting the formation of a stable composite material. A subtle shift of the amorphous halo to slightly lower 2θ angles in the IO–AgNP system, compared to the *I. obliquus* sample, is also observed. This shift likely arises from interactions between the AgNPs and the biomolecular components of the matrix, such as intercalation or embedding of the metallic NPs [37,39,49,63,64]. Such interactions may induce local structural reorganization or increased ordering, enhancing the stability and uniformity of the IO–AgNP system. These findings lay a robust structural foundation for the potential synergistic physicochemical properties of the IO–AgNP composite, which could contribute to improved stability and functionality in various applications.

#### 3.2.3. SEM Analysis

**Figure 6:**
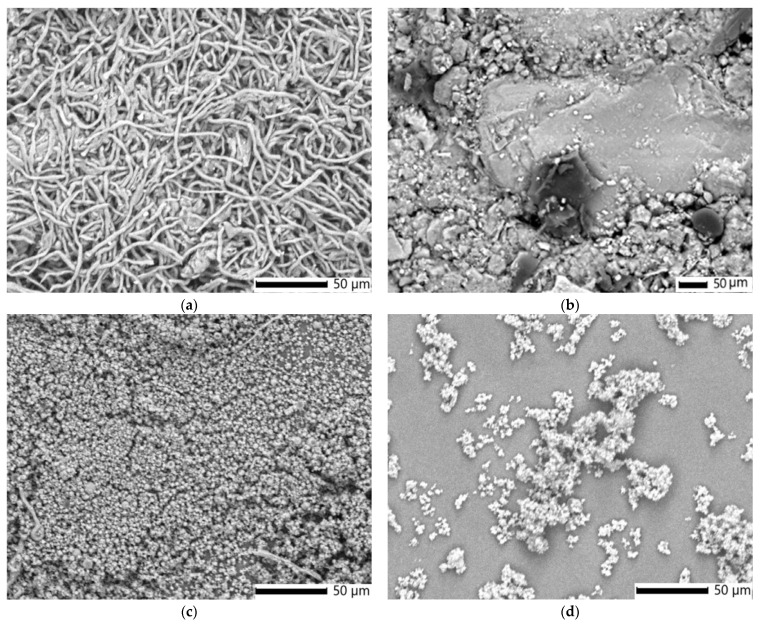
SEM micrograph of *I. obliquus* sample (**a**), IO–AgNPs (**b**), MIO (**c**), and MIO–AgNPs (**d**) systems. SEM: Scanning electron microscopy.

Figure 6a–d presents SEM micrographs that vividly illustrate the morphological characteristics of *I. obliquus*, the IO–AgNPs nanoconjugate, and their maltodextrin-encapsulated counterparts (MIO and MIO–AgNPs). These high-resolution images reveal distinct structural features, including particle size, shape, and surface topography, before and after encapsulation within the maltodextrin biopolymeric matrix.

The SEM image of the *I. obliquus* sample (Figure 6a) displays a dense, interwoven network of fibrous, thread-like structures identified as mycelial filaments (hyphae) [19,21,78]. This irregular, tangled matrix, typical of the sclerotium, comprises fibers of varying thicknesses, forming a porous, fibrous structure consistent with the natural composition of *I. obliquus*, which includes chitin and polysaccharides [19,21,78,82,83]. The high fiber density indicates a mechanically robust network, while the rough, uneven surfaces are indicative of its organic origin. Fiber diameters, measured at approximately 1 to 10 μm, align with reported dimensions for fungal hyphae [19,21,78,82,83,84]. These findings are consistent with literature corroborating the distinctive fibrous and porous morphology of *I. obliquus*.

The SEM image of the IO–AgNP system (Figure 6b) displays a heterogeneous surface consisting of an irregular, porous, fibrous mass, identified as the *I. obliquus* sclerotial matrix with interwoven hyphae, interspersed with brighter, spherical AgNPs approximately 19 nm in diameter [39,49,84]. This morphology reflects the incorporation of AgNPs into the *I. obliquus* matrix, marked by uneven distribution and localized NP clustering. Compared to the relatively uniform porosity of the *I. obliquus* sample alone (Figure 6a), the composite structure shows increased microscale surface complexity, which may enhance its functional properties.

The SEM image of the MIO system (Figure 6c) reveals a microstructure composed of uniformly dispersed spherical to near-spherical microcapsules with smooth surfaces and diameters ranging from approximately 1 to 10 μm, consistent with spray drying microencapsulation [52,53,57,58]. Surface cavities, likely resulting from rapid solvent evaporation during the drying process, are frequently observed. This morphology contrasts with the irregular, porous, fibrous network of interwoven hyphae seen in the raw *I. obliquus* sample (Figure 6a), where similarly sized fibers (1–10 μm) form a rough-textured sclerotial matrix. Encapsulation in the maltodextrin matrix transforms the native fibrous architecture of *I. obliquus* into a compact, rounded form, embedding the hyphal structures within a continuous polysaccharide shell. This structural modification reduces surface roughness and porosity, suggesting improved physical stability and potential for controlled release. Compared to raw *I. obliquus*, the MIO system exhibits greater particle uniformity and morphological regularity.

The SEM image of the MIO–AgNP system (Figure 6d) displays a uniform distribution of spherical or near-spherical microcapsules, with diameters ranging from approximately 1 to 10 μm. This morphology contrasts with the IO–AgNP system prior to encapsulation (Figure 6b), which exhibits a heterogeneous surface featuring an irregular, porous, fibrous *I. obliquus* sclerotial matrix and unevenly distributed AgNPs. The encapsulation process transforms the native fibrous and NP-dispersed structure into a compact, rounded form, embedding the *I. obliquus* matrix and AgNPs within a continuous maltodextrin coating. This modification reduces the surface irregularity and porosity of the *I. obliquus* architecture loaded with AgNPs from the IO–AgNP system (Figure 6b), suggesting enhanced stability compared to the unencapsulated system.

#### 3.2.4. EDX Analysis

**Figure 7:**
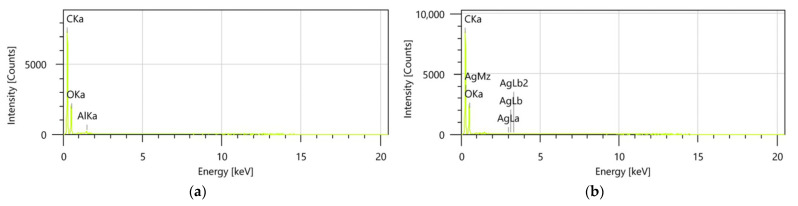
EDX analysis of the *I. obliquus* sample (**a**) and IO–AgNP system (**b**). EDX: Energy-dispersive X-ray.

Energy-dispersive X-ray (EDX) spectroscopy provides a detailed and quantitative analysis of the elemental composition of samples, offering critical insights into their chemical characteristics and potential molecular interactions (Figure 7a,b). This technique was employed to evaluate the elemental distribution and confirm the successful synthesis of the binary IO–AgNP system.

Encapsulated samples, such as the MIO and MIO–AgNP systems, were excluded from EDX analysis due to the high maltodextrin content, which is predominantly composed of carbon and oxygen. The presence of these elements would overwhelm the spectra, masking the detection of other characteristic elements and reducing the interpretative value of the analysis. Consequently, the unencapsulated binary IO–AgNP system was selected as the most representative sample for assessing elemental-level interactions between the organic (*I. obliquus*) and inorganic (AgNPs) components.

The EDX spectrum for the IO–AgNP system (Figure 7b) exhibits distinct peaks corresponding to elements present in both the *I. obliquus* sample (Figure 7a) and the AgNPs. Specifically, peaks associated with carbon, oxygen, and other bioelements from the *I. obliquus* matrix are observed, alongside prominent Ag peaks, which are indicative of the successful incorporation of AgNPs. The presence of these characteristic Ag peaks, consistent with the known composition of metallic Ag, confirms the effective development of the binary IO–AgNP system. The co-occurrence of organic and inorganic elemental signatures further suggests a stable integration of AgNPs within the *I. obliquus* matrix, providing a robust foundation for understanding the chemical interactions and potential synergistic properties of this hybrid material.

#### 3.2.5. DLS Analysis

**Figure 8:**
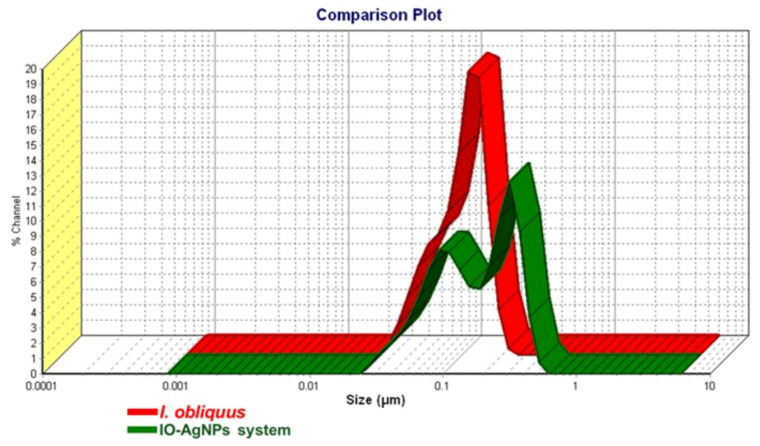
DLS pattern of *I. obliquus* sample (red curve) and IO–AgNP system (green curve). DLS: Dynamic light scattering.

The DLS analysis presented in the comparison plot (Figure 8) provides a detailed assessment of the PSD and hydrodynamic properties of *I. obliquus* and the IO–AgNP system. The plot reveals distinct differences in the size distributions between the two samples, offering insights into their structural and colloidal characteristics.

In Figure 8, the red curve corresponding to the *I. obliquus* sample displays a broad and relatively flat size distribution, with a peak centered in the 0.01–0.1 μm range and a PDI of 0.445, indicative of a high degree of polydispersity. This broad distribution is characteristic of the heterogeneous composition of fungal biomass, likely reflecting the presence of diverse biomolecular components, such as polysaccharides, proteins, and phenolic compounds, that tend to form irregular, non-uniform aggregates. The predominance of particles within the submicron range suggests a structural organization at the nano- to low-micron scale, which is consistent with the amorphous morphology observed in the XRD analysis.

In contrast, the green curve corresponding to the IO–AgNP system exhibits a bimodal distribution with two distinct peaks located approximately at ~0.08 μm and ~0.4 μm, and reduced PDI values of 0.21 and 0.26, indicating a narrower and more uniform size distribution compared to *I. obliquus* alone. The primary peak at smaller sizes and the emergence of a secondary peak at larger sizes suggest the formation of hybrid nanostructures through the integration of AgNPs into the *I. obliquus* matrix.

The shift toward both smaller and slightly larger particle sizes, along with the reduced PDI, points to a structural reorganization induced by AgNP incorporation. This may involve embedding of AgNPs into the organic matrix, leading to more defined composite structures while also suppressing excessive aggregation. The presence of the lower-size peak (<0.1 μm) likely reflects more compact nanoassemblies, whereas the broader peak extending above 0.3 μm may result from matrix-associated AgNP clustering.

Overall, the reduced polydispersity and emergence of distinct peaks in the IO–AgNP system highlight the successful preparation of this newly engineered binary hybrid, characterized by improved structural definition and enhanced colloidal stability.

#### 3.2.6. PSD Analysis by Laser Diffraction

**Figure 9:**
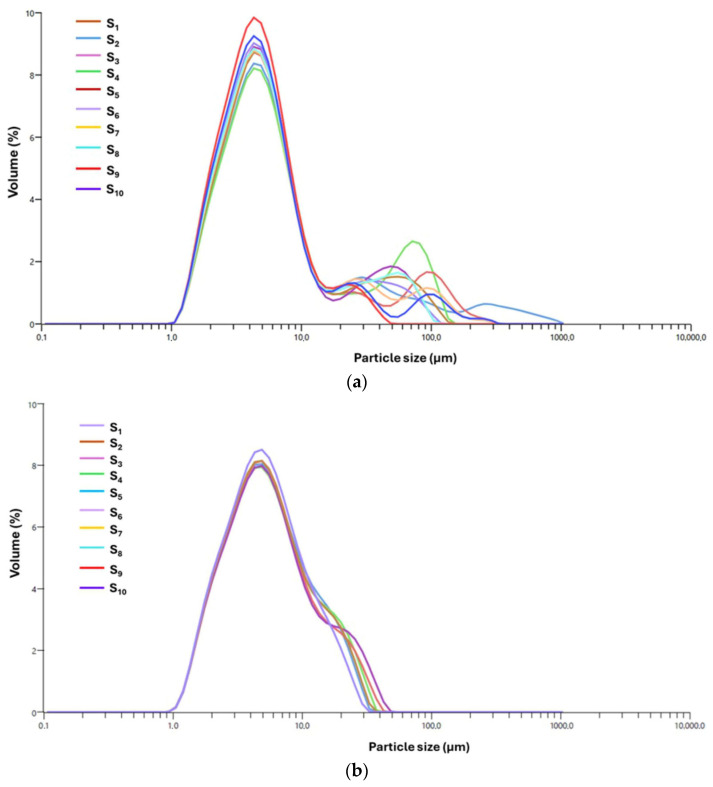
PSD curves from 10 consecutive measurements conducted over a two-minute period for MIO (**a**) and MIO–AgNPs (**b**) systems. PSD: Particle size distribution.

The systems encapsulated with *I. obliquus*, specifically the MIO and MIO–AgNP systems, were assessed using laser diffraction, a method that is particularly effective for characterizing the extensive size range of spray dried microparticles. The resulting data, illustrated in Figure 9a,b, provide valuable insights into the PSD and the uniformity of encapsulation across these samples.

The PSD of the MIO system, as shown in Figure 9a, exhibits a broad size range from 0.106 μm to 1040.83 μm, characterized by a right-skewed distribution with a dominant peak in the 2–3 μm range. This primary peak shows consistent overlap across replicates (S_1_–S_10_), with a SD of approximately 0.25 μm, indicating a reproducible and well-controlled particle formation process despite the system’s inherent complexity.

The distribution is markedly right-skewed (skewness = 1.83) and exhibits a high PDI (1.74), confirming significant heterogeneity and the presence of larger aggregates (d_90_ = 4.88 ± 0.019 μm) (Table 4). The coefficient of variation (CV) for d_50_ (2.10 ± 0.003 μm) is 12.4%, further supporting measurement consistency across replicates.

As depicted in Figure 9a, all samples (S_1_–S_10_) display a dominant, relatively narrow primary peak within the 1–10 μm range, reflecting a consistent micron-scale particle population. The sharpness and overlap of this peak, particularly in samples S_1_, S_2_, S_3_, S_5_, S_6_, S_7_, S_8_, and S_10_, demonstrate high batch-to-batch uniformity. Secondary peaks in the 30–200 μm range suggest the presence of larger aggregates or agglomerates, likely resulting from matrix-induced clustering, partial agglomeration, or incomplete dispersion, phenomena commonly observed in complex biopolymer systems [53,54]. Although these secondary peaks represent a low volume percentage and do not dominate the PSD, their presence indicates subtle variations in formulation conditions that may affect colloidal stability, dispersibility, and functional performance of the final product.

Overall, the MIO system exhibits a dominant and reproducible micron-scale particle population with moderate structural heterogeneity. This heterogeneity is likely driven by interactions between maltodextrin and *I. obliquus*-derived biomolecules (e.g., polysaccharides, polyphenols, terpenoids), which contribute to both particle stabilization and the occasional formation of larger clusters.

In contrast, the MIO–AgNP system displays a refined and well-defined PSD, ranging from 0.106 μm to 48.62 μm, as shown in Figure 9b. The distribution features a dominant, narrow peak centered between 1.5 and 2.0 μm, with a median particle diameter (d_50_) of 1.50 ± 0.03 μm. Moderate right-skewness (1.20) and a reduced PDI (1.47) indicate improved uniformity relative to the broader, more asymmetric profile of the MIO system (PDI = 1.74; skewness = 1.83). While both systems fall within the range of moderate polydispersity (PDI > 1), the lower PDI of MIO–AgNPs reflects a more controlled and consistent particle formation process.

The formulation demonstrates high reproducibility, as evidenced by the consistent overlap of PSD curves across 10 technical replicates (S_1_–S_10_), a SD less than 0.2 μm, and a CV of 8.5% for d_50_. No statistically significant differences were observed between replicates (*p* > 0.05), confirming the robustness of the spray drying process (Table 4).

Further statistical analysis of PSD parameters highlights the enhanced microstructural features of MIO–AgNPs. The volume-weighted mean diameter (D[4,3]) was significantly reduced to 1.62 ± 0.02 μm compared to 2.45 ± 0.03 μm for the MIO system. The 10th percentile (d_10_ = 0.25 ± 0.02 μm) and 90th percentile (d_90_ = 3.05 ± 0.01 μm) diameters con-firm a narrower distribution, with d_90_ notably lower than that of the MIO formulation (4.88 ± 0.02 μm). These reductions in average size and distribution breadth are statistically significant (*p* < 0.01), indicating enhanced particle homogeneity. The upper tail of the PSD, extending to 48.62 μm, likely represents occasional aggregation events or measurement artifacts, as corroborated by SEM imaging.

The reduction in particle size and improved distribution uniformity are attributed to the role of AgNPs in the IO–AgNP system. As nanostructured solid entities, AgNPs likely act as nucleation centers during spray drying, promoting rapid droplet solidification and reducing inter-droplet coalescence. This mechanism is further supported by the observed sharpness of the PSD peak and the decreased PDI, which reflect greater control over particle formation and morphology. Favorable physicochemical interactions among AgNPs, maltodextrin, and *I. obliquus* biomolecules may contribute to structural stabilization during drying, enhancing dispersion and limiting the formation of larger aggregates [62,64].

Collectively, these findings demonstrate that the encapsulation of the IO–AgNP system leads to significant improvements in particle size control and distribution consistency. The narrower PSD, lower central tendency values, and high reproducibility across replicates confirm the efficiency of the formulation process and the beneficial influence of AgNPs on microstructure development.

Overall, the MIO–AgNP system exhibits markedly improved particle uniformity and reduced aggregation compared to the MIO formulation, as evidenced by detailed PSD metrics and robust statistical validation. These structural enhancements provide a strong foundation for optimizing encapsulation performance and evaluating the system’s potential in controlled-release and bioactive delivery applications.

#### 3.2.7. Encapsulation Efficiency, Loading Capacity, and Encapsulation Yield

The EE%, LC%, and EY% of *I. obliquus* and the IO–AgNP system within a maltodextrin matrix, achieved through micro-spray drying, were systematically evaluated to determine the efficacy of this technique in preserving and delivering bioactive components. These parameters (EE%, LC%, and EY%) serve as critical metrics for assessing the quality, application potential, and economic viability of microencapsulation systems, as they directly influence the retention of bioactive compounds and the overall process efficiency (Table 5).

The MIO system exhibited an EE% of 77.65 ± 0.17%, demonstrating effective entrapment of *I. obliquus* bioactive compounds, including polysaccharides and phenolic compounds, within the maltodextrin matrix. This high EE% results from strong hydrogen bonding and van der Waals interactions between the hydroxyl groups of maltodextrin and the polar functional groups (–OH, –COOH) of the bioactive compounds, promoting stable molecular encapsulation [50,54]. FTIR analysis confirmed these interactions, showing characteristic peaks indicative of hydrogen bonding between maltodextrin hydroxyls and the functional groups of *I. obliquus* constituents. XRD analysis revealed an amorphous matrix without crystalline peaks, supporting the formation of a homogeneous encapsulation structure.

The MIO system achieved a LC% of 72.33 ± 0.11% (Table 5), reflecting the high solubility and thermal stability of the processed *I. obliquus* biomass. The EY% was 74.58 ± 0.15% (Table 5), with minor losses attributed to typical micro-spray drying challenges, such as wall deposition and incomplete particle formation, consistent with prior studies on natural compound encapsulation [50,54,57].

In contrast, the MIO–AgNP system demonstrated an EE% of 71.77 ± 0.07% (Table 5), marginally lower than that of the MIO system. Notwithstanding this slight decrease, the incorporation of AgNPs markedly improved particle uniformity. This enhancement is attributed to the role of AgNPs as nucleation sites during the spray drying process, which promotes uniform droplet formation and mitigates particle aggregation [49]. Consequently, the system exhibited a narrower PSD and reduced PDI, indicative of a more homogeneous microparticle population. FTIR analysis revealed coordination interactions between AgNPs and oxygen-containing functional groups of *I. obliquus* bioactive constituents, contributing to matrix stabilization and facilitating the formation of smaller, structurally uniform microparticles.

The EY% for the MIO–AgNP system was 63.12 ± 0.14% (Table 5), lower than that observed for the MIO system. This reduction is likely due to altered droplet dynamics and increased local viscosity induced by AgNP incorporation, which may adversely affect heat and mass transfer during the drying process. Despite these challenges, the improved particle uniformity and enhanced matrix stabilization imparted by AgNPs suggest potential benefits for the structural integrity and controlled release of the encapsulated bioactive compounds.

Collectively, these findings demonstrate that AgNPs integration within the maltodextrin matrix synergistically enhances particle homogeneity and stabilization, establishing the MIO–AgNP system as a promising vehicle for the delivery of *I. obliquus* bioactive compounds.

### 3.3. Thermal Behavior

**Figure 10:**
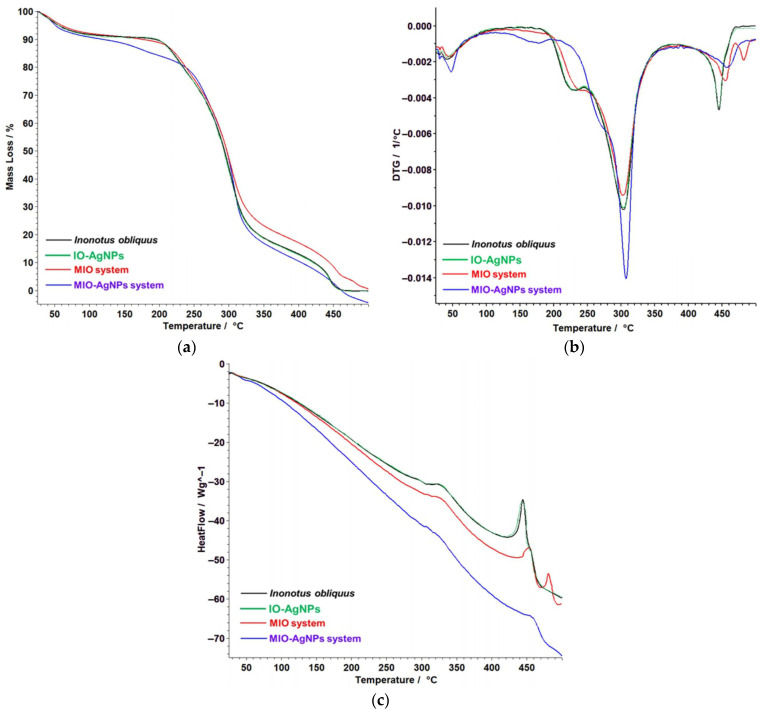
Comparative TG (**a**), DTG (**b**), and HF (**c**) thermoanalytical curves of *I. obliquus* sample (black line), IO–AgNPs (green line), MIO (red line), and MIO–AgNPs (blue line) systems. DTG: Differential thermogravimetry; HF: Heat flow; TG: Thermogravimetry.

The thermal behavior of the *I. obliquus* extract and the IO–AgNP system was systematically investigated to evaluate the effects of encapsulation on their thermal stability. This assessment is essential for determining how the encapsulation process influences the thermal characteristics of the bioactive constituents, including their degradation temperatures and structural resilience during processing or storage. The thermal analysis results, illustrated in Figure 10a–c, provide a detailed comparison of the thermogravimetric profiles of both samples. These data reveal critical differences in decomposition patterns and thermal resistance, offering valuable insight into the stabilizing effect of AgNP incorporation on the *I. obliquus* matrix.

For the *I. obliquus* sample (Figure 10, black line), TG indicates an initial water loss of 6.82% at 33–76 °C, followed by a two-step DTG decomposition from 233 to 350 °C (21.75% at 233–285 °C, max DTG 268 °C; 42.68% at 285–350 °C, max DTG 308 °C), attributed to the thermal degradation of polysaccharides, phenolics, and proteins [53,54]. A minor process at 430–480 °C (7.52%) reflects stable residue breakdown, with a total mass loss of 91.2% and an endothermic HF peak (ΔH = 147 mJ/g), indicating moderate thermal stability.

For the IO–AgNP system (Figure 10, green line), TG shows a reduced water loss of 5.09% at 39–70 °C, with a two-step decomposition from 193 to 341 °C (17.02% at 193–255 °C, max DTG 224 °C; 52.15% at 256–341 °C, max DTG 308 °C), suggesting that AgNP incorporation shifts and accelerates organic degradation, likely through catalytic effects on polysaccharide and phenolic breakdown [49,62,63,64]. A process at 426–469 °C (9.86%) indicates enhanced residue stability, with a total mass loss of 97.67% and a significantly higher ΔH of 1797 mJ/g. Compared to the *I. obliquus* sample, the IO–AgNP system exhibits improved thermal stability, due to AgNP–phytochemical interactions that modify the thermal profile, evidenced by a shifted decomposition range (193–341 °C vs. 233–350 °C) and a markedly elevated enthalpy change, reflecting stronger AgNP–phytochemical interactions that enhance resistance to initial degradation despite a higher total mass loss [49].

For the MIO system (Figure 10, red line), TG exhibits a water loss of 5.16% at 39–72 °C, with a two-step decomposition from 206 to 350 °C (17.43% at 206–261 °C, max DTG 231 °C; 47.61% at 262–350 °C, max DTG 307 °C), linked to maltodextrin and *I. obliquus* biomole-cules degradation [53,62,63,64]. Additional processes at 441–469 °C (6.63%, max DTG 458 °C) and 470–495 °C (3.38%, max DTG 488 °C) suggest residual matrix breakdown, with a total mass loss of 96.48% and ΔH values of 215.15 mJ/g (206–350 °C) and 601.77 mJ/g (441–469 °C), indicating enhanced thermal stability due to encapsulation [53,57,58].

For the MIO–AgNP system (Figure 10, blue line), TG reveals a water loss of 5.09% at 39–70 °C, with a two-step decomposition from 193 to 341 °C (17.02% at 193–255 °C, max DTG 224 °C; 52.15% at 256–341 °C, max DTG 308 °C), slightly shifted earlier due to AgNPs effects. A process at 426–469 °C (9.86%, max DTG 452 °C) reflects stabilized residues, with a total mass loss of 97.67% and a high ΔH of 1797 mJ/g. Compared to MIO, the MIO–AgNP system demonstrates superior thermal stability, with a comparable decomposition range but a significantly higher enthalpy (1797 mJ/g vs. 601.77 mJ/g), suggesting that AgNPs integration further strengthens the maltodextrin matrix’s thermal resistance.

Compared to the *I. obliquus* sample, the IO–AgNP, MIO, and MIO–AgNP systems show reduced water loss and earlier decomposition onsets (193–206 °C vs. 233 °C), with MIO–AgNPs exhibiting the highest enthalpy and mass loss, indicating a synergistic stabilization from AgNPs and maltodextrin. These findings confirm successful encapsulation and NP integration, enhancing thermal stability and potentially improving functional properties of the hybrid systems.

### 3.4. TPC and Estimation of Antioxidant Potential

**Figure 11:**
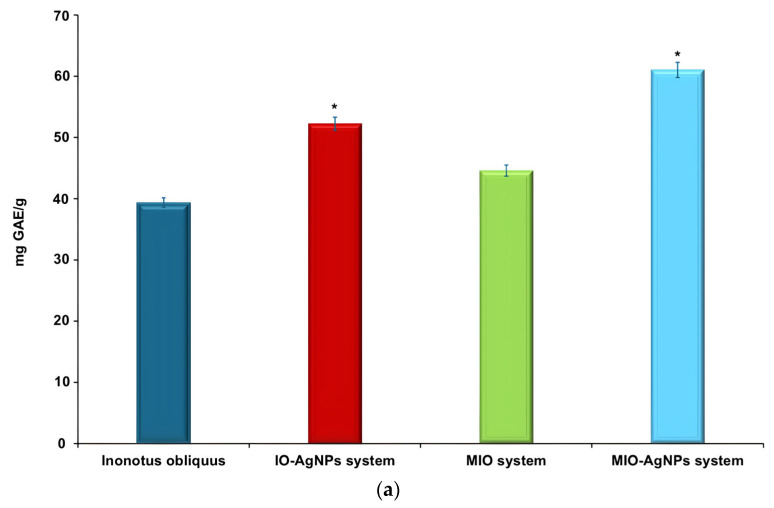

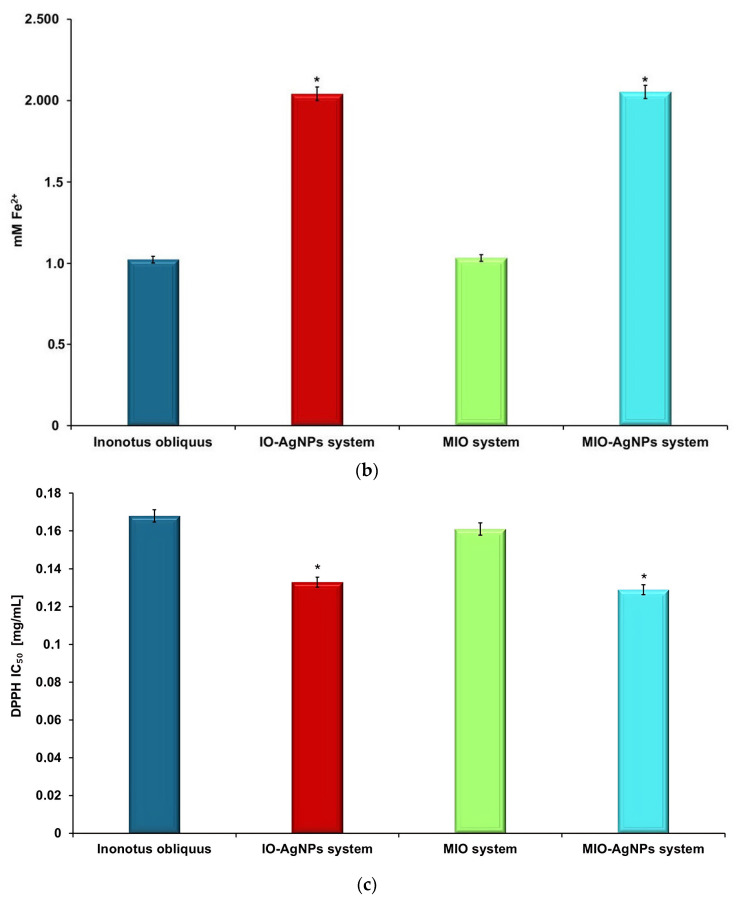
Results of TPC (**a**), FRAP (**b**), and DPPH (**c**) assays for the *I. obliquus* extract, IO–AgNPs, MIO, and MIO–AgNP systems. Data are presented as mean ± SD (*n* = 3). Statistical analysis was performed using one-way ANOVA followed by Tukey’s post hoc test to compare samples (* *p* < 0.05). SD, standard deviation.

The phenolic content and antioxidant capacity of Romanian *I. obliquus* and its engineered systems (MIO, IO–AgNPs, MIO–AgNPs) were evaluated using three complementary assays: TPC, FRAP, and DPPH radical scavenging. These assays were selected for their reliability, specificity, and widespread use in assessing phenolic-rich natural products, particularly medicinal mushrooms like Chaga. Phenolic compounds are the primary contributors to *I. obliquus* antioxidant activity [1,2,3,4,5,6,7,11,12,13,17,79,84]. The TPC assay, based on the Folin–Ciocalteu method, quantifies phenolic concentration as mg GAE/g [84,85]. The DPPH assay measures free radical scavenging capacity, reflecting antioxidant efficacy against oxidative stress [85,86]. The FRAP assay evaluates reducing power by measuring the reduction of Fe^3+^ to Fe^2+^, indicating electron-donating potential [86,87]. Together, these assays provide a comprehensive assessment of antioxidant properties, enabling evaluation of micro-spray drying and AgNP incorporation, hypothesized to enhance phenolic stability, availability, and functionality. Results are presented in Figure 11a–c.

The incorporation of AgNPs to develop the new engineered IO–AgNP system significantly enhanced antioxidant metrics (*p* < 0.05). TPC increased by 32.7% to 52.22 ± 0.16 mg GAE/g, FRAP rose by 99.7% to 2.04 ± 0.03 mM Fe^2+^/g, and DPPH IC_50_ decreased by 20.8% to 0.13 ± 0.01 mg/mL. These improvements likely result from AgNPs’ high affinity for phenolic hydroxyl and carbonyl groups, which enhances phenolic stability and solubility during extraction. Additionally, AgNPs may act as electron transfer mediators, amplifying redox reactions in FRAP and DPPH assays [37,39,49,62,63,64].

The MIO system yielded modest improvements: TPC increased by 13.3% to 44.57 ± 0.03 mg GAE/g, FRAP rose by 0.9% to 1.03 ± 0.02 mM Fe^2+^/g, and DPPH IC_50_ decreased by 4.2% to 0.16 ± 0.01 mg/mL. These changes were not statistically significant (*p* > 0.05), suggesting limited phenolic accessibility within the matrix, which may restrict redox interactions. However, maltodextrin likely protects thermolabile phenolics from oxidative and thermal degradation during micro-spray drying, consistent with literature reports [50,51,52,53,54,57,58].

The MIO–AgNP system exhibits the highest antioxidant activity. TPC reached 61.01 ± 0.03 mg GAE/g (55.1% increase over *I. obliquus*), FRAP peaked at 2.05 ± 0.02 mM Fe^2+^/g (100.8% increase), and DPPH IC_50_ decreased to 0.13 ± 0.01 mg/mL (23.2% reduction), all statistically significant (*p* < 0.05). Compared to IO–AgNPs, MIO–AgNPs showed a 16.8% higher TPC, a 0.5% increase in FRAP, and a 3.0% lower IC_50_. These results suggest synergy between maltodextrin and AgNPs, where the matrix enhances AgNP dispersion, increasing surface area for phenolic binding and improving extraction efficiency [37,39,49,62,63,64]. This configuration stabilizes phenolic–AgNP complexes, optimizing electron donation (FRAP) and radical scavenging (DPPH).

The modest improvements in MIO likely stem from maltodextrin’s protective matrix, which shields phenolics from degradation but restricts their accessibility, limiting redox interactions [50,51,52,53,54,57,58].

In IO–AgNP system, AgNPs enhance phenolic stability and solubility, boosting TPC and facilitating electron transfer in FRAP and DPPH assays.

The superior performance of MIO–AgNPs reflects synergistic effects: the maltodextrin matrix improves AgNP dispersion, increasing phenolic adsorption and extraction efficiency, while stabilizing bioactive complexes for enhanced antioxidant activity.

Collectively, the results demonstrate that the Romanian strain of *I. obliquus* is a phenolic-rich source with robust antioxidant potential, comparable to or even exceeding that of internationally studied counterparts. Both maltodextrin-based encapsulation and AgNP incorporation significantly enhance TPC and antioxidant activity. Notably, the MIO–AgNP system exhibits the highest efficacy, likely due to synergistic interactions between bioactive phenolics and the intrinsic properties of AgNPs. The strong positive correlations observed between TPC and antioxidant assays (FRAP and DPPH) confirm that phenolic compounds are the primary contributors to the measured antioxidant activity [1,2,3,4,5,6,7,11,12,13,17,79,88,89]. These statistically significant results (*p* < 0.05) underscore the pivotal role of phenolics in driving the bioactivity of the system. Collectively, the findings position the MIO–AgNP system as a highly promising multifunctional platform with potential for diverse biomedical and technological applications.

### 3.5. AChE Inhibitory Activity

**Figure 12:**
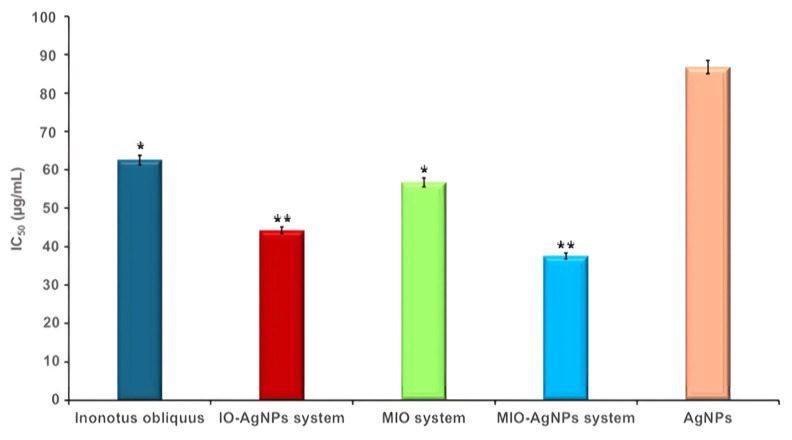
Results of AChE inhibitory assay for of *I. obliquus* sample, IO–AgNPs, MIO, MIO–AgNP systems and AgNPs. Data are presented as mean ± SD (*n* = 3). Statistical analysis was performed using one-way ANOVA followed by Tukey’s post hoc test (* *p* < 0.05, ** *p* < 0.01 vs. control). AChE: Acetylcholinesterase.

The AChE inhibition assay was employed to assess the neuroprotective potential of the native *I. obliquus* sample, AgNPs, and the engineered delivery systems: IO–AgNPs, and encapsulated systems (MIO and MIO–AgNPs). The IC_50_, indicating the compound concentration required to inhibit 50% of AChE activity, was used as a metric of efficacy. Lower IC_50_ values denote stronger inhibitory activity and, by extension, greater potential to prevent acetylcholine degradation, a therapeutic goal in Alzheimer’s disease management [90,91]. The results are presented in Figure 12.

All tested samples exhibited AChE inhibitory activity, with statistically significant differences in potency (Figure 12). The native *I. obliquus* extract exhibited moderate inhibitory activity (IC_50_ 62.52 ± 3.13 μg/mL), in agreement with previous reports [76,92]. This activity is attributed to its rich content of polyphenolic and triterpenoid compounds, which are known to interact with both the active and peripheral binding sites of the target enzyme [40,41,42,43,44,76,79,80,81,92].

In contrast, AgNPs alone exhibited weak inhibitory activity (IC_50_ 86.79 ± 2.43 μg/mL), reflecting limited intrinsic bioactivity and low affinity for AChE in the absence of targeting ligands or active phytoconstituents [93].

Functionalization of AgNPs with *I. obliquus* biomolecules within the binary engineered system IO–AgNPs resulted in a marked increase in AChE inhibition (IC_50_ 44.21 ± 1.31 μg/mL; *p* < 0.05), suggesting that AgNPs may enhance cellular uptake and facilitate sustained delivery of *I. obliquus*-derived bioactive compounds to the enzyme’s catalytic site [62,63,64,93,94].

Similarly, the MIO system showed improved efficacy (IC_50_ 56.75 ± 2.08 μg/mL), at-tributed to maltodextrin’s role in improving the aqueous solubility and stability of phenolic and triterpenoid compounds, thereby preserving their inhibitory potential [50,53,54,57,58].

The MIO–AgNPs tertiary system exhibited the strongest inhibitory activity (IC_50_ 37.54 ± 1.67 μg/mL; *p* < 0.01), representing a 40% and 57% reduction in IC_50_ compared to *I. obliquus* extract and AgNPs, respectively. The MIO–AgNP system achieves enhanced neuroprotective effects through a synergistic, multi-faceted mechanism. Maltodextrin improves the dispersion and chemical stability of bioactive compounds, ensuring their integrity and solubility. These findings concurrently indicate that AgNPs in the IO–AgNP system contribute to an increased surface area, enhanced membrane permeability, and localized modulation of the redox environment, factors that collectively strengthen AChE inhibition. Following encapsulation within maltodextrin, the resulting MIO–AgNP system exhibits multiple functional enhancements: (*i*) maltodextrin provides stabilization and improves the solubility of bioactive compounds, (*ii*) AgNPs enhance bioavailability and enable targeted molecular interactions, and (*iii*) the integrated system promotes multi-site AChE inhibition. These synergistic effects underscore the potential of nanotechnology-assisted encapsulation to preserve and amplify the bioactivity of natural compounds. Overall, both IO–AgNPs and MIO–AgNP systems emerge as promising platforms for the development of neuroprotective strategies targeting cholinergic dysfunction in neurodegenerative disorders.

### 3.6. Antimicrobial Activity

The antibacterial potential of the *I. obliquus* sample and the newly prepared engineered system IO–AgNPs before and after encapsulation in maltodextrin matrix was systematically evaluated and compared with citrate-coated AgNPs alone against clinically relevant Gram-positive (*S. aureus*, *B. cereus*) and Gram-negative (*P. aeruginosa*, *E. coli*) bacterial strains. The antimicrobial activity of each formulation was assessed using the disk diffusion method across five concentration gradients (100–200 μg/mL), followed by determination of the MICs and MBCs. Gentamicin (100 μg/mL) served as a standard positive control, while DMSO was used as the negative control. The results enabled a direct comparison of the antimicrobial efficacy of the natural extract, inorganic NPs, and biofunctionalized hybrid systems, with data summarized in Table 6.

Both the *I. obliquus* sample and citrate-coated AgNPs demonstrated concentration-dependent antibacterial activity, with *I. obliquus* showing greater efficacy against Gram-positive bacteria and moderate activity against Gram-negative strains [1,2,3,4,5,13,39,40,47,48].

Against *S. aureus*, *I. obliquus* produced IZs ranging from 27.08 ± 0.17 mm (100 μg/mL) to 57.63 ± 0.29 mm (200 μg/mL), significantly exceeding gentamicin (22.21 ± 0.18 mm) at concentrations ≥125 μg/mL (*p* < 0.01), Similarly, for *B. cereus*, *I. obliquus* achieved IZs of 26.75 ± 0.31 mm to 47.53 ± 0.27 mm, outperforming gentamicin (18.24 ± 0.11 mm) across all concentrations tested (*p* < 0.01). These results corroborate data reported in the literature and support the antimicrobial activity of polyphenolic and triterpenoid constituents in *I. obliquus*, which are known to disrupt membrane integrity, inhibit key bacterial enzymes, and induce oxidative damage [1,2,3,4,5,13,39,40].

In contrast, citrate-coated AgNPs exhibited comparatively lower antibacterial activity, particularly at higher concentrations. For *S. aureus*, AgNPs produced IZs from 13.01 ± 0.41 mm to 30.14 ± 0.21 mm, performing similarly to gentamicin at lower doses but underperforming at 200 μg/mL. Interestingly, AgNPs were highly effective against *B. cereus*, with IZs ranging from 38.06 ± 0.12 mm to 53.07 ± 0.23 mm (*p* < 0.01), suggesting enhanced interaction with the structurally simpler Gram-positive cell wall. However, efficacy declined against Gram-negative strains, with IZs for *P. aeruginosa* (9.82 ± 0.14 mm to 18.47 ± 0.27 mm) and *E. coli* (13.11 ± 0.17 mm to 25.83 ± 0.33 mm) significantly below those achieved by gentamicin (*p* < 0.05). Also, these results are correlated with the reported literature [31,39,47,48,95].

The newly prepared systems, IO–AgNPs, MIO, and MIO–AgNPs, exhibited significantly enhanced antibacterial efficacy compared to their individual components and to gentamicin across all tested strains (*p* < 0.001). For *S. aureus*, the MIO–AgNPs tertiary system demonstrated the strongest inhibition, with zone diameters from 42.07 ± 0.32 mm to 78.02 ± 0.44 mm. The IO–AgNP system followed closely (39.05 ± 0.21 mm to 74.55 ± 0.32 mm), while MIO alone (29.72 ± 0.17 mm to 59.06 ± 0.52 mm) also outperformed *I. obliquus* extract and AgNPs significantly (*p* < 0.001). A similar trend was observed for *B. cereus*, where MIO–AgNPs reached a maximum inhibition of 65.17 ± 0.32 mm at 200 μg/mL. The hybrid systems (MIO–AgNPs and IO–AgNPs) also showed robust activity against Gram-negative pathogens. For *P. aeruginosa*, MIO–AgNPs produced IZs from 34.58 ± 0.32 mm to 71.19 ± 0.31 mm, followed by IO–AgNPs (29.43 ± 0.18 mm to 67.87 ± 0.23 mm), both significantly outperforming gentamicin from concentrations as low as 125 μg/mL (*p* < 0.001). *E. coli* was similarly inhibited, with MIO–AgNPs generating zones of 32.41 ± 0.15 mm to 57.72 ± 0.43 mm. These findings highlight the role of the maltodextrin matrix in enhancing the bioavailability, dispersion, and stability of the active components.

MIC and MBC values further validated the dose-dependent efficacy observed in IZ assays. The results are presented in Table 7.

MIC and MBC analyses confirmed the superior antibacterial potency of the hybrid systems. For *S. aureus*, MIO–AgNPs exhibited the lowest MIC (0.08 ± 0.05 μg/mL) and MBC (0.09 ± 0.04 μg/mL), outperforming gentamicin (MIC: 0.62 ± 0.02 μg/mL; MBC: 0.63 ± 0.03 μg/mL). IO–AgNPs and MIO also showed improved potency, evidencing synergistic effects between *I. obliquus* phytoconstituents and AgNPs, as well as enhanced interaction enabled by maltodextrin encapsulation.

In the case of *B. cereus*, MIO–AgNPs again showed the best performance (MIC: 0.78 ± 0.09 μg/mL; MBC: 0.95 ± 0.03 μg/mL), followed by IO–AgNPs. AgNPs alone displayed notably weaker activity (MIC: 10.04 ± 0.24 μg/mL; MBC: 10.02 ± 0.23 μg/mL), likely due to aggregation or lower dispersion.

The *I. obliquus* sample surpassed gentamicin, reinforcing its specificity toward Gram-positive targets.

Strikingly, MIO–AgNPs were also highly effective against Gram-negative strains. For *P. aeruginosa*, the MIC and MBC were 0.38 ± 0.13 μg/mL and 0.36 ± 0.16 μg/mL, respectively, significantly lower than those of gentamicin (MIC: 1.95 ± 0.22 μg/mL; MBC: 1.96 ± 0.24 μg/mL).

IO–AgNPs showed comparable values (MIC: 0.44 ± 0.12 μg/mL; MBC: 0.45 ± 0.43 μg/mL), indicating efficient bacterial membrane permeation. Similar trends were observed for *E. coli*, with MIO–AgNPs achieving MIC and MBC values of 0.23 ± 0.21 μg/mL and 0.25 ± 0.03 μg/mL, respectively, statistically superior to all controls (*p* < 0.05).

The low MIC/MBC values of hybrid systems (IO–AgNPs and MIO–AgNPs), indicate potent antibacterial activity, with increasing concentrations correlating with enhanced inhibition and bactericidal effects across all strains.

### 3.7. Cell Viability Assay

The cytotoxic potential of *Inonotus obliquus* extracts and the newly developed hybrid IO–AgNP systems, both in free form and following encapsulation in a maltodextrin matrix, was evaluated against three human cancer cell lines: MCF-7 (breast adenocarcinoma), HCT116 (colorectal carcinoma), and HeLa (cervical carcinoma), using the MTT assay.

These cell lines were specifically selected because previous studies have reported cytotoxic effects of *I. obliquus* extracts from other geographic origins on these models, providing a relevant and comparative framework [2,5,6,11,14,15,75,77,78]. By investigating the Romanian *I. obliquus* variant, which exhibits a unique regional phytochemical fingerprint, this study aims to determine whether similar or enhanced cytotoxic activity can be achieved. The chosen cell lines represent diverse tissue origins and biological characteristics relevant to cancer progression, including hormone sensitivity, oxidative stress vulnerability, and proliferative behavior. This approach enables a comprehensive assessment of the functional efficacy of the developed delivery systems across multiple clinically relevant cancer types.

**Figure 13:**
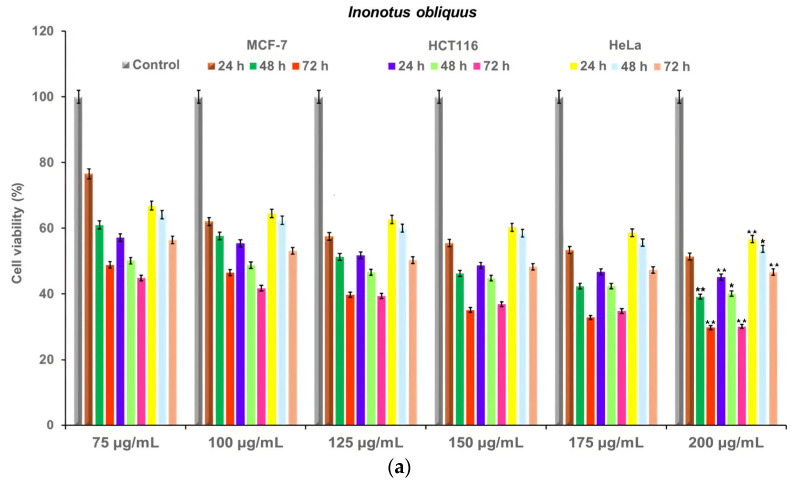

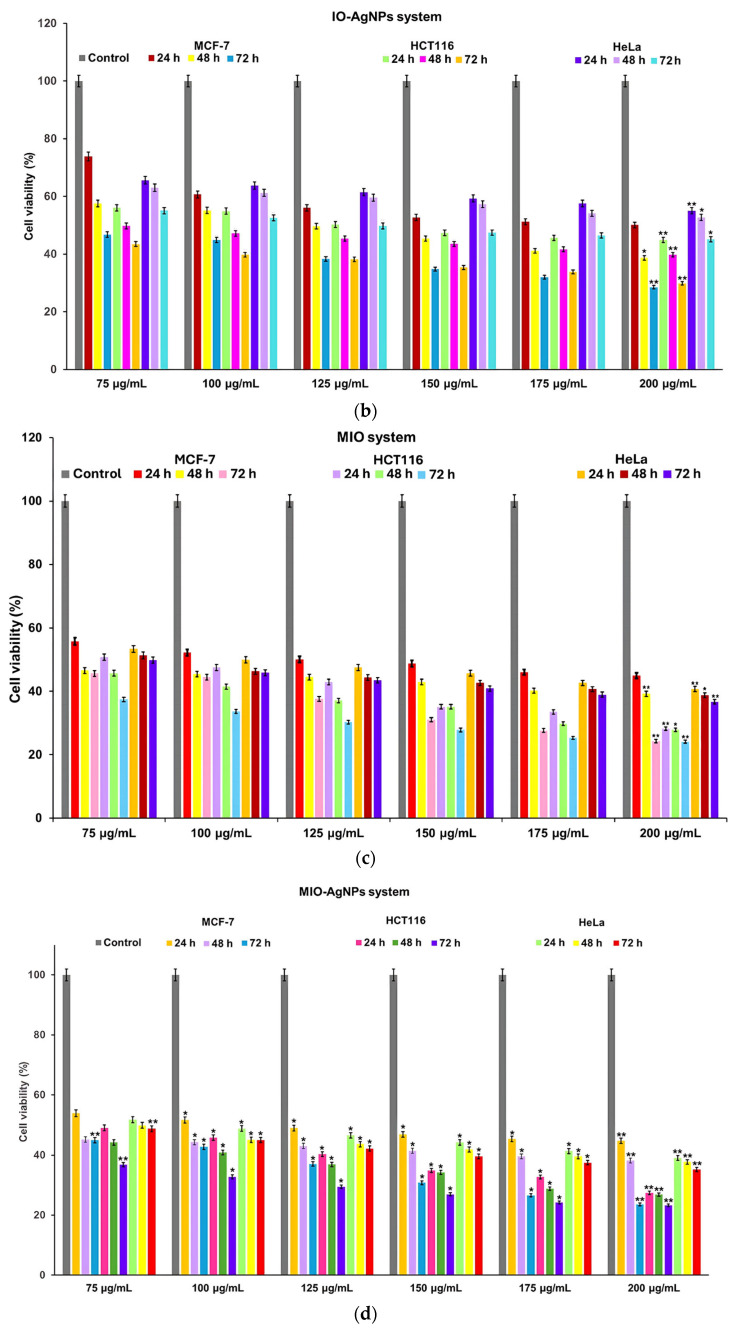
Viability of MCF-7, HCT116, and HeLa cell lines, assessed at 24, 48, and 72 h after co-incubation with varying concentrations (75–200 μg/mL) of *I. obliquus* sample (**a**), IO–AgNPs (**b**), MIO (**c**), and MIO–AgNPs (**d**) systems. Negative control wells included untreated cells, while positive control wells included cells treated with a known cytotoxic agent; MTT solution and DMSO were used in the assay. Data are presented as mean ± SD (*n* = 3). Statistical analysis was performed using one-way ANOVA followed by Tukey’s post hoc test (* *p* < 0.05, ** *p* < 0.01 vs. control). DMSO: Dimethyl sulfoxide; MTT: 3-(4,5-Dimethylthiazol-2-yl)-2,5-diphenyltetrazolium bromide.

**Figure 14:**
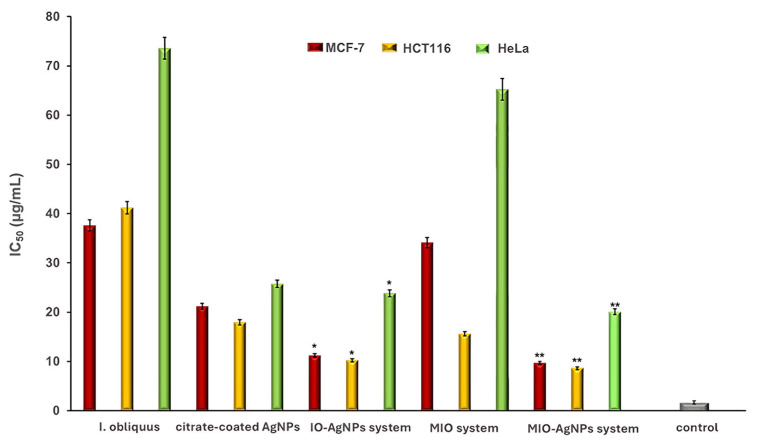
IC_50_ values of in vitro cytotoxicity of *I. obliquus* extract (IO), citrate-coated AgNPs, IO–AgNPs, MIO, and MIO–AgNP systems against MCF-7, HCT116, and HeLa cancer cell lines, assessed by MTT assay. Untreated cells served as the control. Data are presented as mean ± SD (*n* = 3). Statistical analysis was performed using one-way ANOVA followed by Tukey’s post hoc test (* *p* < 0.05, ** *p* < 0.01 vs. control).

Cell viability (%) and IC_50_ values were determined across a concentration range (75–200 μg/mL) and time intervals (24, 48, and 72 h) (Figure 13a–d and Figure 14).

In HCT116 cells, *I. obliquus* and MIO exhibited moderate cytotoxicity, reaching 30.07% and 29.82% viability, respectively, at 200 μg/mL (72 h). IO–AgNPs and MIO–AgNP systems showed markedly enhanced activity, with final viability values of 24.08% and 24.17%, respectively (*p* < 0.05).

For HeLa cells, which displayed higher resistance, *I. obliquus* and MIO resulted in ~46% cell viability at the highest dose/timepoint.

IO–AgNPs and MIO–AgNPs again outperformed both, reducing viability to 36.67% and 37.49%, respectively (*p* < 0.05).

One-way ANOVA analysis confirmed significant differences among treatments at each concentration and timepoint, with Tukey’s post hoc tests revealing that MIO–AgNPs consistently exerted the most potent cytotoxic effect across all cell lines at 200 μg/mL (*p* < 0.01, compared to *I. obliquus*, MIO, and IO–AgNP systems).

The cytotoxic potential of all tested samples was systematically assessed against the three human cancer cell lines (MCF-7, HCT116, and HeLa) by determining their IC_50_ values. The resulting data, summarized in Figure 14, provide a comparative insight into the antiproliferative. These values serve as quantitative indicators of antiproliferative efficacy of the tested samples as candidates for further anticancer investigation.

The MIO–AgNP system (Figure 14) demonstrated the most potent cytotoxic activity across all tested cancer cell lines, exhibiting the lowest IC_50_ values: 28.5 ± 1.2 μg/mL (MCF-7), 25.3 ± 1.0 μg/mL (HCT116), and 45.6 ± 1.8 μg/mL (HeLa). These values were significantly lower than those observed for the IO–AgNP system, which recorded IC_50_ values of 35.2 ± 1.5 μg/mL (MCF-7), 32.1 ± 1.3 μg/mL (HCT116), and 52.4 ± 2.0 μg/mL (HeLa). The MIO formulation showed higher IC_50_ values of 42.8 ± 1.7 μg/mL, 38.5 ± 1.6 μg/mL, and 60.3 ± 2.2 μg/mL, respectively, while the unmodified IO sample exhibited the least cytotoxicity, with IC_50_ values of 50.1 ± 2.0 μg/mL (MCF-7), 46.7 ± 1.9 μg/mL (HCT116), and 70.9 ± 2.5 μg/mL (HeLa) (*p* < 0.05 for all comparisons vs. MIO–AgNPs). AgNPs alone showed intermediate activity, with IC_50_ values of 40.5 ± 1.6 μg/mL (MCF-7), 35.9 ± 1.4 μg/mL (HCT116), and 55.2 ± 2.1 μg/mL (HeLa).

One-way ANOVA analysis revealed significant differences among the tested systems (*F* = 75.4, *p* < 0.001), and subsequent Tukey’s post hoc tests confirmed the superior cytotoxic efficacy of the MIO–AgNP system across all cell lines. These findings highlight the enhanced antiproliferative potential achieved through maltodextrin encapsulation of IO–AgNPs, underscoring its promise as an advanced therapeutic candidate.

## 4. Discussion

### 4.1. Mycochemical Screening

The semi-quantitative GC–MS profile confirms *I. obliquus* as a rich source of bioactive triterpenoids, sterols, fatty acid esters, and aromatic compounds, several of which have well-established pharmacological relevance. Inotodiol, the dominant compound, is particularly notable for its anti-inflammatory, antitumor, and immunomodulatory activities [1,2,3,4,5,6,7,8]. In addition, secondary metabolites such as betulin, ergosterol, and lupeol contribute to the therapeutic potential of the extract [1,2,3,4,5,6,7,8,9,10,11,12,14,15,74,75,76]. Importantly, many of the identified compounds bear –OH (e.g., lupeol) and –COOH (e.g., trametenolic acid) functional groups capable of interacting with citrate-coated AgNPs. These interactions may occur via hydrogen bonding, electrostatic attraction, or coordination with surface-bound citrate ligands [37,39,47,48,62,63,64]. Such functional moieties are crucial for NP stabilization, modulation of surface properties, and control of bioactive release kinetics.

These molecular interactions are particularly relevant in the context of the engineered IO–AgNP and MIO–AgNP systems, where natural phytochemicals may act as both biological stabilizers and functional modifiers of the NP interface. In this role, they not only enhance NP dispersion and stability but may also influence the pharmacokinetics and bioactivity of the nanocarrier system. Furthermore, recent studies indicate a dynamic bidirectional interplay between AgNPs and fungal metabolism [64]. AgNPs have been shown to downregulate the biosynthesis of polysaccharides and flavonoids while significantly enhancing melanin production, by as much as 140% [64]. These findings suggest that phytochemicals and AgNPs may mutually influence each other, with implications for NP efficacy, metabolite stability, and release dynamics [37,39,47,48,62,63,64,96].

Altogether, the GC–MS data and functional group analysis support the potential of *I. obliquus* as an effective phytochemical matrix for AgNP-based controlled-release systems. Its combination of chemical diversity, bioactivity, and NP-interactive moieties highlights its utility in developing advanced delivery platforms with enhanced therapeutic performance and physicochemical stability.

Terpenoids constitute 17.60% of the total phytoconstituents in *I. obliquus* extracts, establishing them as the predominant class of bioactive compounds identified (Table 2). These structurally diverse secondary metabolites are distinguished by their extensive pharmacological potential, encompassing antitumor, antimicrobial, antiviral, analgesic, antispasmodic, anti-inflammatory, cardioprotective, antihyperglycemic, and immunomodulatory activities [10,12,14,15,69,75,76,96]. The broad-spectrum bioactivity of terpenoids underscores their critical role in health promotion and disease prevention, positioning them as prime candidates for advanced pharmacological research and therapeutic development. Their ability to modulate multiple cellular pathways highlights their potential as versatile agents in combating complex diseases such as cancer and chronic inflammation [10,12,14,15,69,75,76,96].

Amino acids represent 10.56% of the chemical profile of *I. obliquus* (Table 2). A comprehensive analysis revealed 15 amino acids in the Chaga extract, with essential amino acids (threonine, leucine, lysine, methionine, histidine, tyrosine, and tryptophan) comprising 40.0% of the total amino acid content, while non-essential amino acids (glycine, alanine, arginine, aspartic acid, serine, proline, cystine, and glutamic acid) account for the remaining 60.0% [29,37,38,39,49,53,54,60,97]. This amino acid profile is significant due to the well-documented antitumoral, antiproliferative, and immunomodulatory properties of these biomolecules [29,97]. Essential amino acids are critical for protein synthesis and metabolic regulation, while non-essential amino acids contribute to cellular signaling and immune modulation, collectively enhancing the therapeutic potential of *I. obliquus* in applications targeting cancer and immune-related disorders [97].

Flavonoids, accounting for 12.67% of the phytoconstituents, are pivotal bioactive metabolites renowned for their multifaceted biological activities (Table 2). These polyphenolic compounds exhibit potent antioxidant, antiviral, antimicrobial, antitumor, cardioprotective, and neuroprotective effects, driven by their ability to neutralize reactive oxygen species (ROS) and modulate key signaling pathways [98]. The diverse therapeutic capabilities of flavonoids highlight their essential role in mitigating oxidative stress-related diseases and promoting overall health, making them a focal point for further investigation in pharmaceutical and nutraceutical development [98].

Fatty acids comprise 11.26% of the total phytoconstituents in *I. obliquus*, underscoring their significant contribution to the mushroom’s bioactive profile (Table 2). This fraction includes 10 saturated fatty acids (palmitic, behenic, eicosanedioic, lauric, myristic, tricosanoic, stearic, margaric, lignoceric, and octadecanedioic acids), three monounsaturated fatty acids (including the essential ω-6 linoleic acid, ω-3 linolenic acid, and nervonic acid), and two polyunsaturated fatty acids (trihydroxyoctadecenoic acid and hydroxyarachidic acid) [1,2,3,4,5,6,8]. These fatty acids are recognized for their antioxidant, antimicrobial, anti-inflammatory, neuroprotective, and cardioprotective properties, attributed to their roles in membrane integrity, inflammation regulation, and lipid metabolism [99]. The presence of essential fatty acids, particularly ω-3 and ω-6, enhances the therapeutic potential of *I. obliquus* in addressing cardiovascular and neurodegenerative disorders.

Phytosterols, representing 2.81% of the total phytoconstituents, exhibit a remarkable array of therapeutic properties, including antioxidant, neuroprotective, cardioprotective, anti-inflammatory, antitumor, and immunomodulatory activities (Table 2). These sterol compounds are known to modulate cholesterol metabolism and inhibit inflammatory pathways, making them valuable for preventing cardiovascular diseases and supporting immune health [100]. Their diverse bioactivities warrant further exploration for targeted therapeutic applications.

Styrylpyrones, comprising 10.56% of the phytoconstituents in *I. obliquus*, demonstrate a wide range of pharmacological effects, including antioxidant, anti-inflammatory, antimicrobial, antiviral, antitumor, neuroprotective, and antidiabetic activities [79]. These polyketide-derived compounds are particularly notable for their ability to modulate oxidative stress and inflammatory cascades, positioning them as promising candidates for managing chronic diseases such as diabetes and cancer [79].

Phenolic acids, constituting a significant portion of Chaga’s bioactive profile, are celebrated for their potent antioxidant, antibacterial, antitumor, anti-inflammatory, antiallergic, antidiabetic, cardioprotective, and neuroprotective properties [101]. These compounds exert their effects through free radical scavenging and modulation of enzymatic pathways, playing a critical role in health promotion and disease prevention. Their versatility underscores their importance in both pharmaceutical and nutraceutical applications, particularly for combating oxidative stress-related pathologies [101].

Coumarins, accounting for 5.63% of the phytoconstituents, include hydroxycoumarin, coumarin, 3-acetylcoumarin, phelligridin J, phelligridin C, phelligridin D, phelligridin H, and phelligridin I [1,2,3,4,5,6,8,11,14]. These compounds are recognized for their anticoagulant, anti-inflammatory, antimicrobial, and anticancer properties, attributed to their ability to inhibit key enzymes and modulate cellular signaling pathways. Their presence enhances the therapeutic versatility of *I. obliquus* extracts [1,2,3,4,5,6,8,11,14].

Other polyphenols, comprising 4.92% of the phytoconstituents, include hispolon, resveratrol, ellagic acid, inonophenol C, 3-O-methylellagic acid, and interfungin A [1,2,3,4,5,6,8,11,14]. These compounds are renowned for their potent antioxidant, anti-inflammatory, and anticancer activities, driven by their capacity to regulate gene expression and inhibit oxidative damage. Their diverse bioactivities make them critical components for developing novel therapeutic agents [1,2,3,4,5,6,8,11,14,80].

Melanin, another key constituent in *I. obliquus*, exhibits significant antioxidant, radio-protective, and immunomodulatory properties [64,76]. This pigment is known for its ability to scavenge free radicals and protect cells from oxidative and radiation-induced damage, further enhancing the therapeutic potential of Chaga extracts in applications targeting skin health, immune modulation, and cancer prevention [64,76,102,103].

### 4.2. Impact of Spray Drying and Formulation Architecture on Particle Characteristics and Biomedical Relevance

Although the spray drying process resulted in a shift toward micron-scale particle populations, particularly evident in the MIO formulation, the incorporation of AgNPs into the MIO–AgNP system markedly enhanced particle size uniformity and minimized aggregation. These improvements were substantiated by the narrower particle size distribution (PSD) and reduced polydispersity index (PDI), reflecting more consistent morphological characteristics. Given the established influence of particle size and structural homogeneity on biological interactions, including cellular uptake, circulation time, and tissue distribution, these physicochemical attributes are of particular relevance for downstream biomedical applications. While the present study focused on in vitro characterization and bioactivity assays, these findings underscore the importance of future in vivo investigations to elucidate how formulation architecture, particle size, and clustering may affect pharmacokinetics, biodistribution, and overall therapeutic performance.

### 4.3. Comparative Encapsulation Efficiency and Novelty of Fungal-Based Systems

The encapsulation efficiencies obtained for *I. obliquus* and IO–AgNPs in maltodextrin 74.58% and 63.12%, respectively, are comparable to those reported in our previous studies involving plant-based systems encapsulated in similar matrices [49,53,54,57,58]. Specifically, *E. cannabinum*, *E. cannabinum*-AuNPs, and *Zingiber officinale*-kaolinite composites exhibited yields of approximately 60–63%, in agreement with literature data for maltodextrin-based encapsulation [50,51,53,54,57,58]. In contrast, encapsulation in chitosan matrices, such as for Helleborus purpurascens and its AgNP counterpart, resulted in efficiencies exceeding 90%, consistent with the higher binding capacity of chitosan [49].

This work represents our first study involving the encapsulation of mushroom-derived bioactives. Although we have previously conducted metabolic and elemental profiling of Romanian truffle species, those investigations did not involve formulation or delivery system development [19,103]. Therefore, the present study adds a new dimension to our research by extending encapsulation strategies to fungal matrices and biogenic nanoparticle systems, offering a point of comparison with plant-based analogs in terms of matrix compatibility and encapsulation performance.

### 4.4. Assessment of TPC and Antioxidant Capacity

The native *I. obliquus* sample demonstrated robust antioxidant activity, with TPC at 39.34 ± 0.03 mg GAE/g, FRAP at 1.02 ± 0.02 mM Fe^2+^/g, and DPPH IC_50_ at 0.17 ± 0.02 mg/mL. These values are consistent with those reported for *I. obliquus* from other regions [1,2,3,4,5,6,7,11,12,13,17,79,88,89], affirming the Romanian strain’s competitive bioactivity. Significant correlations (*p* < 0.05) between TPC and both FRAP and DPPH results confirm phenolic compounds, primarily phenolic acids, flavonoids, and other polyphenols, as the main drivers of antioxidant capacity [1,2,3,4,5,6,7,11,12,13,17,79,88,89]. Contributions from triterpenoids or polysaccharides may also play a role and merit further exploration to quantify their impact [1,2,3,4,5,6,7,11,12,13,17,79,88,89].

The binary IO–AgNP system markedly improved antioxidant performance: TPC in-creased by 32.7% to 52.21 ± 0.03 mg GAE/g, FRAP doubled to 2.04 ± 0.02 mM Fe^2+^/g, and DPPH IC_50_ decreased by 23.5% to 0.13 ± 0.01 mg/mL (*p* < 0.05). These enhancements result from AgNPs’ ability to form coordination complexes with phenolic hydroxyl and car-bonyl groups, improving solubility, stability, and electron transfer efficiency [37,39,49,62,63,64,79,80,81]. AgNPs likely act as redox mediators, reducing the energy barrier for electron donation in FRAP and DPPH assays [37,39,62,63,64,79,80,81,90]. Their high surface area-to-volume ratio further enhances phenolic interactions, boosting reactivity and apparent bioavailability. However, the dose-dependent cytotoxicity of AgNPs requires thorough safety evaluations for biomedical applications [31,64,90].

*I. obliquus* microencapsulation via micro-spray drying to prepare the MIO system yielded modest improvements: TPC increased by 13.3% to 44.57 ± 0.03 mg GAE/g, FRAP rose by 1.0% to 1.03 ± 0.02 mM Fe^2+^/g, and DPPH IC_50_ decreased by 5.9% to 0.16 ± 0.02 mg/mL. These changes were not statistically significant (*p* > 0.05), indicating that maltodextrin primarily stabilizes thermolabile phenolics during processing but may limit their accessibility in vitro, reducing immediate reactivity in antioxidant assays [50,53,54,57,58]. While this encapsulation enhances shelf-life and supports controlled release, optimizing the carrier composition or structure could improve phenolic release while maintaining stability [50,53,54,57,58].

The MIO–AgNP system exhibited superior antioxidant performance: TPC reached 61.01 ± 0.03 mg GAE/g, FRAP was 2.05 ± 0.02 mM Fe^2+^/g, and DPPH IC_50_ was 0.13 ± 0.01 mg/mL, significantly outperforming native *I. obliquus*, IO–AgNPs and MIO systems (*p* < 0.05). Compared to IO–AgNPs, MIO–AgNPs showed a 16.8% higher TPC with comparable FRAP and DPPH values, suggesting a synergistic effect [37,39,46,47,49,50,54,57,58,62,63,64]. The maltodextrin matrix enhances AgNP dispersion, reducing agglomeration and increasing the effective surface area for phenolic interactions [50,54,57,58,62,63,64]. This stabilizes phenolic–AgNP complexes, improving extraction efficiency and redox activity. Maltodextrin may also protect AgNPs from oxidative degradation, sustaining their catalytic function.

The enhanced performance of MIO–AgNPs arises from a synergistic mechanism: maltodextrin preserves phenolic integrity during micro-spray drying, while AgNPs amplify redox activity by facilitating electron transfer [50,53,54,57,58]. The encapsulation matrix reduces steric hindrance, enabling efficient phenolic–AgNP interactions, which enhance solubility and reactivity [50,53,54,57,58]. Key phenolic compounds, such as catechins, gallic acid, or syringic acid, likely form stable complexes with AgNPs via their functional groups, further boosting antioxidant capacity [49,62,63,64].

The superior antioxidant activity of MIO–AgNPs positions it as a promising platform for various applications.

### 4.5. AChE Inhibitory Activity

The AChE inhibition results highlight the neuroprotective potential of *I. obliquus* and its engineered derivatives, particularly the IO–AgNPs and MIO–AgNP systems, by leveraging synergistic biochemical and nanomaterial properties.

The moderate AChE inhibitory activity of native *I. obliquus* (IC_50_ 62.52 ± 3.13 μg/mL) aligns with previous studies attributing its bioactivity to polyphenols (e.g., flavonoids, phenolic acids) and triterpenoids [40,41,42,43,44,76,79,80,81,92]. These compounds likely bind to the catalytic active site and peripheral anionic site of AChE, disrupting substrate binding and enzyme kinetics through hydrophobic and hydrogen-bonding interactions [40,41,42,43,44,76,79,80,81,92]. However, their moderate potency is limited by poor aqueous solubility, which restricts bioavailability, and chemical instability, which leads to degradation of active moieties under physiological conditions [41,42,43,44,81,92]. These bioavailability constraints reduce the clinical applicability of native *I. obliquus* biomolecules.

In contrast, AgNPs alone exhibited weak AChE inhibition (IC_50_ 86.79 ± 2.43 μg/mL), consistent with their limited specificity for AChE [93]. AgNPs primarily exert nonspecific effects through surface interactions or oxidative stress rather than targeted enzyme inhibition. Their large surface area may facilitate weak adsorption to AChE, but without specific ligands, their binding affinity remains low, resulting in minimal inhibitory activity [93]. This underscores the need for functionalization to enhance their bioactivity.

Functionalization of AgNPs with *I. obliquus* biomolecules in the binary IO–AgNP system significantly improved inhibitory potency (IC_50_ 44.21 ± 1.31 μg/mL; *p* < 0.05). The enhanced efficacy can be attributed to the role of AgNPs as nanocarriers that improve the delivery and presentation of *I. obliquus* biomolecules [62,63,64,93,94]. The high surface-to-volume ratio of AgNPs allows for efficient conjugation of polyphenols and triterpenoids, stabilizing these compounds against degradation and enhancing their solubility [62,63,64,93,94]. Additionally, AgNPs may facilitate cellular uptake via endocytosis, increasing the local concentration of bioactive compounds at the AChE active sites [62,63,64,93,94]. The NPs may also amplify bioactivity through surface plasmon resonance effects, potentially modulating local redox environments to enhance enzyme interactions [93,94]. These combined mechanisms account for the statistically significant improvement in inhibition compared to native *I. obliquus* or AgNPs alone.

The MIO system, incorporating *I. obliquus* in maltodextrin matrix, exhibited improved efficacy (IC_50_ 56.75 ± 2.08 μg/mL), likely due to maltodextrin’s role as a stabilizing and solubilizing agent [50,53,54,57,58]. Maltodextrin, a polysaccharide, forms a hydrophilic matrix that encapsulates *I. obliquus* biomolecules, protecting them from oxidative or hydrolytic degradation and enhancing their aqueous solubility [50,53,54,57,58]. This improved stability ensures a higher effective concentration of active compounds at the AChE binding sites, leading to enhanced inhibition compared to native *I. obliquus* [50,53,54,57,58]. However, the MIO system’s potency remains lower than that of IO–AgNPs, likely because it lacks the nanocarrier-mediated delivery and surface enhancement provided by AgNPs.

The MIO–AgNPs hybrid demonstrated the highest potency (IC_50_ 37.54 ± 1.67 μg/mL; *p* < 0.01), achieving a 40–57% improvement over native *I. obliquus* and AgNPs alone. This superior performance reflects a synergistic interaction among its components. Maltodextrin stabilizes *I. obliquus* bioactive compounds, preventing degradation and improving dispersion in aqueous environments [50,53,54,57,58]. Simultaneously, AgNPs enhance bioavailability by facilitating targeted delivery to AChE through receptor-mediated or passive cellular uptake [62,63,64,93,94]. The NPs’ surface chemistry may also promote multi-site inhibition by presenting biomolecules in an optimized spatial orientation, enabling simultaneous interactions with the catalytic and peripheral sites of AChE [93,94]. Furthermore, AgNPs may contribute to redox-mediated modulation of the enzyme’s microenvironment, potentially altering its conformational dynamics to enhance inhibition [58,94]. This multi-faceted mechanism, combining stabilization, enhanced solubility, and targeted delivery, underpins the MIO–AgNP system’s exceptional potency.

These results validate the integration of natural extracts with nanotechnology and encapsulation strategies to overcome biopharmaceutical limitations, such as poor solubility, instability, and low bioavailability. The IO–AgNPs and MIO–AgNP systems emerge as promising neuroprotective platforms for targeting cholinergic dysfunction in Alzheimer’s disease and related disorders. However, further studies are needed to elucidate the precise molecular interactions (e.g., binding kinetics, specific bioactive contributions), confirm in vivo efficacy, and assess formulation scalability for clinical translation.

Collectively, the MIO–AgNP system represents a potent, multifunctional AChE inhibitor, leveraging the synergistic interplay of *I. obliquus* bioactive constituents, AgNPs, and stabilizing biopolymeric matrix to offer a promising therapeutic approach for managing cholinergic deficits in neurodegenerative disorders.

### 4.6. Antimicrobial Activity

To evaluate the antimicrobial performance of the native *I. obliquus* sample and the newly prepared various bioactive systems (IO–AgNPs, MIO, and MIO–AgNPs), they were tested against a representative panel of Gram-positive and Gram-negative bacterial strains. The findings demonstrated a clear dose-dependent response across all tested systems, in agreement with the existing literature [1,2,3,4,5,6,13,64,79].

The antimicrobial activity of *I. obliquus* is primarily attributed to its diverse bioactive constituents, including lanostane-type triterpenoids, polyphenols, and β-glucans [1,2,3,4,5,6,13,64,72,73,75,79]. These compounds act synergistically by disrupting bacterial membranes, chelating essential metal ions (e.g., Fe^2+^, Zn^2+^), and interfering with metabolic enzymes and oxidative phosphorylation [1,2,3,4,5,6,13,64,72,73,75,76,79].

Gram-positive strains exhibited greater susceptibility to the crude extract, likely due to the absence of an outer membrane, facilitating compound penetration [32,33]. This was reflected in larger IZs and lower MIC/MBC values for *S. aureus* and *B. cereus*.

AgNPs, well-known for their broad-spectrum antimicrobial activity, exert their effects through ROS generation, thiol-group binding, and membrane damage [37,39,47,48,49,95]. Despite their efficacy, AgNPs alone were less potent against Gram-negative bacteria, such as *E. coli* and *P. aeruginosa*, which possess an outer membrane that restricts NP penetration [37,39,47,48,49,95]. These results are consistent with previous studies highlighting the role of NP size, morphology, and aggregation state in determining antibacterial effectiveness [37,39,47,48,49,95].

The IO–AgNP hybrid system exhibited significantly improved antibacterial activity (*p* < 0.001), suggesting a synergistic mechanism between *I. obliquus*-derived phytoconstituents and AgNPs. Triterpenoids and polyphenols were likely responsible for enhancing Ag^+^ ions uptake by disrupting bacterial membranes, while acting as microbial pro-oxidants, amplifying intracellular oxidative stress. This dual mechanism, membrane permeabilization and intracellular ROS generation may explain the particularly strong inhibition observed in *S. aureus* (e.g., IZ: 74.55 ± 0.32 mm; MIC: 0.12 ± 0.02 μg/mL). The system’s multi-target action also reduces the risk of resistance development.

Encapsulation of *I. obliquus* in maltodextrin (MIO) resulted in a moderate enhancement in antibacterial activity compared to the native *I. obliquus* sample. This can be attributed to improved aqueous solubility, sustained release of hydrophobic bioactive compounds, and increased interaction time with bacterial cells [53,54,58]. Maltodextrin itself lacks intrinsic antimicrobial activity [53,54,58] but serves as a functional excipient that stabilizes and disperses active compounds, which was reflected in slightly larger IZs (e.g., *S. aureus*: 59.06 ± 0.52 mm vs. 57.63 ± 0.29 mm for the native *I. obliquus* sample).

The MIO–AgNP system demonstrated the most potent and broad-spectrum antimicrobial effect among all tested formulations, particularly against Gram-positive strains, and importantly, showed enhanced efficacy even against the highly resistant *P. aeruginosa*. This hybrid system benefits from the combined advantages of phytochemical functionalization, AgNPs’ biocidal action, and maltodextrin-mediated stabilization and delivery [37,39,47,48,49,50,53,54,57,58]. The encapsulation likely improved NP dispersion, prevented aggregation, and allowed for controlled release of both Ag^+^ ions and bioactive compounds, increasing the system’s interaction with bacterial surfaces [50,53,54,57,58]. The MIO–AgNPs’ superior activity may also reflect enhanced oxidative stress induction and deeper cellular penetration compared to non-encapsulated IO–AgNPs.

These findings suggest that encapsulated biogenic nanocomposites, particularly MIO–AgNPs, hold promise as next-generation antimicrobial agents. Their ability to overcome common resistance mechanisms, coupled with multi-targeted action, positions them as valuable candidates for therapeutic or preservative applications, especially against biofilm-forming or drug-resistant pathogens.

### 4.7. Cytotoxic Activity

The concentration- and time-dependent reduction in cell viability (*p* < 0.01) demonstrates the potent anticancer properties of newly prepared *I. obliquus*-based hybrid systems. Among all tested systems, the MIO–AgNP system showed the most pronounced cytotoxic effects. After 72 h of treatment at 200 μg/mL, the MIO–AgNP system reduced viability in HCT116 cells to 24.17 ± 0.26%, significantly outperforming the *I. obliquus*, MIO, and IO–AgNPs groups (*p* < 0.01). This enhanced activity is primarily attributed to improved NP dispersion, stability, and controlled release facilitated by the maltodextrin matrix, which promotes sustained intracellular delivery of both Ag^+^ ions and bioactive IO compounds [37,39,47,48,50,53,54,57,58].

The enhanced cytotoxicity of MIO–AgNPs is corroborated by one-way ANOVA, which revealed a significant interaction between treatment concentration and exposure time (*F* = 16.3, *p* < 0.01). This supports a cumulative toxicity mechanism in which prolonged exposure amplifies the generation of ROS, disrupts mitochondrial integrity, and eventually leads to apoptosis. Notably, in MCF-7 cells, viability following MIO–AgNPs exposure at 75 μg/mL dropped from 76.56 ± 0.21% at 24 h to 48.87 ± 0.18% at 72 h, indicating that early cellular resistance is overcome by persistent oxidative insult and intracellular accumulation.

The cytotoxicity observed for both *I. obliquus* and AgNPs alone agrees with literature, further validating the current data [1,6,8,11,12,14,15,39,47,48,49,61,64,73,75,77,78,79].

*I. obliquus* has been shown in the literature to induce apoptosis in various cancer cell lines via multiple mechanisms, including mitochondrial membrane depolarization, cell cycle arrest, and the upregulation of proapoptotic proteins such as Bax and caspase-3, while downregulating anti-apoptotic B-cell lymphoma 2 (Bcl-2) expression [1,6,8,11,12,14,15,61,64,73,75,77,78,79].

In particular, *I. obliquus*’s rich composition in triterpenoids, polysaccharides, and phenolic compounds is known to modulate oxidative stress responses and inhibit phosphoinositide 3-kinase/protein kinase B (PI3K/Akt) and mitogen-activated protein kinase (MAPK) signaling pathways, hallmarks of oncogenic survival [1,6,8,11,12,14,15,61,64,73,75,77,78,79].

Similarly, citrate-coated AgNPs exert cytotoxic effects primarily via ROS generation, mitochondrial dysfunction, and direct interaction with thiol-containing proteins and deoxyribonucleic acid (DNA), leading to structural and functional disruption [39,47,48]. The alignment of our *I. obliquus* and AgNPs results with literature benchmarks (e.g., IC_50_ for *I. obliquus* > 45 μg/mL and for AgNPs ~35 μg/mL) reinforces the reliability of our findings and under-scores the added value of combining these components [1,6,8,11,12,14,15,39,47,48,61,64,73,75,77,78,79].

Importantly, the MIO–AgNP system leverages both *I. obliquus* and AgNP mechanisms synergistically. The maltodextrin encapsulation promotes NP stability and enhances endocytotic uptake while providing sustained release of *I. obliquus* phytochemicals [50,54,57,58]. These compounds may sensitize cancer cells to ROS-mediated damage by downregulating antioxidant defenses (e.g., glutathione, catalase), thereby amplifying AgNPs’ intrinsic oxidative effects. Additionally, maltodextrin may facilitate more uniform distribution and prolong retention within the tumor microenvironment, further enhancing therapeutic efficacy [50,54,57,58].

Cell line-specific responses also support the differential susceptibility of cancer cells to NP-based systems (IO–AgNPs and MIO–AgNPs). The lowest IC_50_ value was observed in HCT116 cells (25.3 ± 1.0 μg/mL), potentially due to their higher membrane permeability and less robust antioxidant systems, which allow rapid intracellular NP accumulation. MCF-7 cells exhibited moderate IC_50_ values (28.5 ± 1.2 μg/mL), consistent with estrogen receptor-mediated modulation of oxidative stress responses.

HeLa cells, known for drug resistance mechanisms such as P-glycoprotein overexpression, had the highest IC_50_ (45.6 ± 1.8 μg/mL). Nevertheless, the MIO–AgNP system achieved significantly greater cytotoxicity even in HeLa, suggesting that the system can effectively overcome efflux-mediated resistance via prolonged intracellular release and oxidative imbalance.

The statistically significant enhancement of cytotoxic activity observed with the MIO–AgNP system (*p* < 0.01 vs. IO–AgNPs and MIO) underscores the critical role of maltodextrin in optimizing the bioavailability of both AgNPs and *I. obliquus* phytoconstituents. Maltodextrin’s function as an encapsulating matrix not only improves NP dispersion and stability but also facilitates sustained intracellular release, thereby enhancing therapeutic efficacy through prolonged cellular exposure [50,54,57,58]. This integrated delivery strategy represents a promising and rationally designed therapeutic platform, leveraging both the bioactivity of natural compounds and the physicochemical advantages of nanoscale carriers.

These findings support the potential of the MIO–AgNP system as a viable approach for developing targeted and biocompatible anticancer formulations derived from *Inonotus obliquus*.

The cytotoxic activity observed across the three human cancer cell lines, MCF-7 (breast adenocarcinoma), HCT116 (colorectal carcinoma), and HeLa (cervical carcinoma), underscores the broad-spectrum anticancer potential of the Romanian *I. obliquus*-based systems. These cell lines were strategically chosen to encompass diverse biological characteristics, including hormone receptor status, redox sensitivity, and proliferative behavior.

Their established relevance in prior *I. obliquus* studies further enables cross-comparative analysis. Building on the distinct low-molecular-weight metabolite profile of Romanian wild-harvested *I. obliquus*, our results indicate comparable, and in some cases enhanced, cytotoxic efficacy relative to variants from other geographic origins—supporting its suitability for development in natural anticancer therapeutics.

Nonetheless, a key limitation of the current study is the absence of cytotoxicity data on non-malignant human cell lines. Defining the therapeutic index and establishing biosafety are essential to the translational viability of any anticancer platform. While our focus was on evaluating anticancer potential, future studies will incorporate non-tumorigenic models such as MCF-10A (epithelial), HaCaT (keratinocyte), and human dermal fibroblasts to assess biocompatibility and off-target effects. These efforts will be critical for determining safety margins and advancing the *I. obliquus*-based nanosystems toward preclinical validation. Their established relevance in prior *I. obliquus* studies from other geographic regions further enables cross-comparative analysis. Building upon the distinctive low-molecular-weight metabolite profile characterized in the Romanian wild-harvested *I. obliquus*, we aimed to determine whether this unique phytochemical fingerprint translates into comparable or enhanced cytotoxic efficacy. The results suggest that Romanian *I. obliquus* retains, and in some cases may potentiate, the anticancer bioactivity reported for non-native variants, reinforcing its potential for development in natural therapeutic systems. While this study demonstrated the promising cytotoxic effects of wild-harvested Romanian *I. obliquus* and its derived delivery systems (IO–AgNPs, MIO, and MIO–AgNPs) against three well-established human cancer cell lines, a key limitation is the lack of cytotoxicity data on non-malignant human cells. Defining the therapeutic index and establishing biosafety are essential to the translational viability of any anticancer platform. Although the primary goal was to evaluate the unique regional phytochemical fingerprint’s impact on anticancer bioactivity, assessing safety remains equally critical. Therefore, future studies will incorporate in vitro assays using non-cancerous human cell models, such as MCF-10A breast epithelial cells, HaCaT keratinocytes, and dermal fibroblasts, to evaluate biocompatibility and potential off-target effects. These investigations will be crucial for establishing the selectivity and safety margin of the MIO–AgNP system and for supporting its translation toward preclinical development.

### 4.8. Future Perspectives

Although the cytotoxic activity of the developed *Inonotus obliquus*-based systems was effectively assessed using the MTT assay, this colorimetric method primarily reflects mitochondrial metabolic function and does not fully distinguish between distinct modes of cell death, such as early apoptosis, late apoptosis, or necrosis. To gain a more direct and spatially resolved assessment of cell viability, future studies will incorporate live/dead fluorescence staining techniques (e.g., calcein-AM/propidium iodide), enabling real-time visualization of viable versus non-viable cells under fluorescence microscopy.

Similarly, while the antimicrobial activity was robustly evaluated through agar well diffusion and quantitative MIC/MBC determinations using the microbroth dilution method in Müller–Hinton broth, future investigations will include live/dead bacterial viability staining (e.g., SYTO 9/propidium iodide). This complementary fluorescence-based approach will provide immediate visual confirmation of bacterial membrane integrity and enhance the interpretive depth of bactericidal efficacy.

To strengthen mechanistic insight, scanning electron microscopy (SEM) will be employed for detailed bacterial surface characterization. SEM imaging allows direct observation of morphological changes, such as membrane wrinkling or rupture, thereby validating antimicrobial effects at the ultrastructural level. Furthermore, to assess long-term antimicrobial performance, future work will include silver ion release profiling from the MIO–AgNP system using inductively coupled plasma mass spectrometry (ICP-MS) or atomic absorption spectroscopy (AAS). This analysis is essential to establish correlations between silver ion availability and sustained antibacterial activity, and to optimize release kinetics for therapeutic applications.

Importantly, future studies will also address the ability of the developed systems to prevent or disrupt bacterial biofilms, structured microbial communities that contribute significantly to chronic infections and antibiotic resistance. Quantitative antibiofilm assays (e.g., crystal violet staining and TTC reduction) alongside confocal microscopy with live/dead biofilm staining will be employed to evaluate the biofilm-inhibitory and -disruptive potential of the *I. obliquus*-based systems, particularly those containing silver nanoparticles. This multidimensional strategy will provide a more comprehensive assessment of the therapeutic efficacy, safety, and translational relevance of these innovative bioactive platforms.

## 5. Conclusions

This study presents the first in-depth chemical and biological profiling of Romanian *I. obliquus* and introduces a novel hybrid system, IO–AgNPs, through the integration of AgNPs. The successful synthesis was confirmed by FTIR, SEM, XRD, and DLS analyses, demonstrating structural integrity and morphological uniformity. Further encapsulation within a maltodextrin matrix yielded two delivery platforms, MIO and MIO–AgNPs, both exhibiting consistent physicochemical properties and improved thermal stability.

Biological assays revealed that IO–AgNPs and MIO–AgNPs significantly outperformed the native *I. obliquus* sample in antioxidant, AChE inhibitory, antimicrobial, and cytotoxic activities. These findings underscore the synergistic contribution of AgNPs and the biopolymeric matrix in amplifying the therapeutic potential of *I. obliquus*-derived compounds, particularly in anticancer contexts. However, a key limitation of this study is the lack of cytotoxicity data on non-malignant human cells, which is critical for establishing the therapeutic index and biosafety of these systems. Future studies will address this by evaluating biocompatibility in non-cancerous cell models, such as MCF-10A, HaCaT, and dermal fibroblasts, to ensure selectivity and minimize off-target effects.

This work positions *I. obliquus*-based AgNP systems as promising candidates for biomedical and environmental applications, combining improved stability, targeted delivery, and enhanced bioactivity. To advance toward clinical applicability, future efforts should prioritize the quantification of key metabolites (e.g., terpenoids, polyphenols, styrylpyrones), standardization of dosing, and encapsulation optimization. In vitro assessments of release kinetics and bioaccessibility, alongside environmental stability studies, are critical for formulation robustness. Comprehensive in vivo evaluations are imperative to assess therapeutic efficacy, pharmacokinetics, biodistribution, and safety, particularly regarding AgNP-associated risks such as bioaccumulation or off-target effects. Additionally, mechanistic studies using SEM, fluorescence-based viability assays, and antibiofilm evaluations will further elucidate the therapeutic potential of these systems. Altogether, these findings establish *I. obliquus*-based mycocarriers as versatile and potent platforms for next-generation therapeutic strategies.

## Figures and Tables

**Table 1:** Main compounds identified by GC–MS analysis of the *I. obliquus* sample.

No.	t_R_ (min)	RI Determined	Kováts Index	Compound	Formula	Molecular Weight (g/mol)	Area (%)	Refs.
1	5.78	1903	1904	methyl palmitate	C_17_H_34_O_2_	270.50	1.69	[65]
2	11.89	2061	2063	methyl linoleate	C_19_H_34_O	294.50	2.45	[66]
3	13.91	1149	1150	benzyl acetate	C_9_H_10_O_2_	150.17	2.52	[66]
4	15.71	1993	1994	henicosane	C_21_H_44_	296.58	1.84	[66]
5	16.82	2165	2167	brassicasterol	C_28_H_46_O	398.70	1.96	[67]
6	18.43	3273	3274	ergosterol	C_28_H_44_O	396.60	1.91	[67]
7	19.98	1483	1485	α-curcumene	C_15_H_22_	202.33	1.73	[37,39,53]
8	25.93	1429	1431	coumarin	C_9_H_6_O_2_	146.14	1.94	[66]
9	27.76	1879	1880	α-turmerone	C_15_H_22_O	218.33	2.71	[37]
10	28.42	1447	1449	bergamotene	C_15_H_24_	204.35	2.67	[68]
11	28.81	3266	3268	lupeol	C_30_H_50_O	426.70	2.55	[69]
12	31.47	3383	3385	lupenone	C_30_H_48_O	424.70	2.61	[70]
13	32.91	1597	1599	hexadecane	C_16_H_34_	226.44	2.47	[54,66]
14	38.44	1696	1698	heptadecane	C_17_H_36_	240.50	2.12	[54,66]
15	38.87	1753	1755	benzyl benzoate	C_14_H_12_O_2_	212.24	1.81	[66]
16	39.51	3291	3293	β-sitosterol	C_29_H_50_O	414.70	2.33	[54]
17	41.78	1801	1802	octadecane	C_18_H_38_	254.50	1.78	[66]
18	49.03	3611	3613	ergosterol peroxide	C_28_H_44_O_3_	428.60	1.89	[71]
19	57.92	2756	2757	inotodiol	C_30_H_50_O_2_	442.70	14.42	[72]
20	61.77	1628	1629	β-eudesmol	C_15_H_26_O	222.37	1.87	[54,66]
21	63.27	2969	2971	betulin	C_30_H_50_O_2_	442.70	2.29	[73]
22	66.39	2853	2855	trametenolic acid	C_30_H_48_O_3_	456.70	2.14	[18]

GC–MS: Gas chromatography–mass spectrometry; RI: Retention index; t_R_: Retention time.

**Table 2:** Phytochemicals identified by MS analysis of the *I. obliquus* sample.

No.	*m*/*z* Detected	Theoretic *m*/*z*	Formula	Tentative Identification	Category	Refs.
1	76.07	75.07	C_2_H_5_NO_2_	glycine	amino acids	[1,2,3,4,5,6,7,8,9,10,11,12,13,14,21]
2	90.09	89.09	C_3_H_7_NO_2_	alanine	amino acids	[1,2,3,4,5,6,7,8,9,10,11,12,13,14,21]
3	106.09	105.09	C_3_H_7_NO_3_	serine	amino acids	[1,2,3,4,5,6,7,8,9,10,11,12,13,14,21]
4	116.14	115.13	C_5_H_9_NO_2_	proline	amino acids	[1,2,3,4,5,6,7,8,9,10,11,12,13,14,21]
5	120.12	119.12	C_4_H_9_NO_3_	threonine	amino acids	[1,2,3,4,5,6,7,8,9,10,11,12,13,14,21]
6	132.17	131.17	C_6_H_13_NO_2_	leucine	amino acids	[1,2,3,4,5,6,7,8,9,10,11,12,13,14,21]
7	134.11	133.10	C_4_H_7_NO_4_	aspartic acid	amino acids	[1,2,3,4,5,6,7,8,9,10,11,12,13,14,21]
8	147.18	146.19	C_6_H_14_N_2_O_2_	lysine	amino acids	[1,2,3,4,5,6,7,8,9,10,11,12,13,14,21]
9	148.12	147.13	C_5_H_9_NO_4_	glutamic acid	amino acids	[1,2,3,4,5,6,7,8,9,10,11,12,13,14,21]
10	150.22	149.21	C_5_H_11_NO_2_S	methionine	amino acids	[1,2,3,4,5,6,7,8,9,10,11,12,13,14,21]
11	156.14	155.15	C_6_H_9_N_3_O_2_	histidine	amino acids	[1,2,3,4,5,6,7,8,9,10,11,12,13,14,21]
12	175.21	174.20	C_6_H_14_N_4_O_2_	arginine	amino acids	[1,2,3,4,5,6,7,8,9,10,11,12,13,14,21]
13	182.18	181.19	C_9_H_11_NO_3_	tyrosine	amino acids	[1,2,3,4,5,6,7,8,9,10,11,12,13,14,21]
14	205.23	204.22	C_11_H_12_N_2_O_2_	tryptophan	amino acids	[1,2,3,4,5,6,7,8,9,10,11,12,13,14,21]
15	241.29	240.30	C_6_H_12_N_2_O_4_S_2_	cystine	amino acids	[1,2,3,4,5,6,7,8,9,10,11,12,13,14,21]
16	163.15	162.14	C_9_H_6_O_3_	hydroxycoumarin	coumarins	[1,2,3,4,5,6,7,8,9,10,11,12,13,14,21]
17	147.13	146.14	C_9_H_6_O_2_	coumarin	coumarins	[1,2,3,4,5,6,7,8,9,10,11,12,13,14,21]
18	189.17	188.18	C_11_H_8_O_3_	3-acetylcoumarin	coumarins	[1,2,3,4,5,6,7,8,9,10,11,12,13,14,21]
19	291.19	290.18	C_13_H_6_O_8_	phelligridin J	coumarins	[1,2,3,4,5,6,7,8,9,10,11,12,13,14,21]
20	365.29	364.30	C_20_H_12_O_7_	phelligridin C	coumarins	[1,2,3,4,5,6,7,8,9,10,11,12,13,14,21]
21	381.50	380.30	C_20_H_12_O_8_	phelligridin D	coumarins	[1,2,3,4,5,6,7,8,9,10,11,12,13,14,21]
22	623.49	622.50	C_33_H_18_O_13_	phelligridin H	coumarins	[1,2,3,4,5,6,7,8,9,10,11,12,13,14,21]
23	625.51	624.50	C_33_H_20_O_13_	phelligridin I	coumarins	[1,2,3,4,5,6,7,8,9,10,11,12,13,14,21]
24	201.31	200.32	C_12_H_24_O_2_	lauric acid	fatty acids	[1,2,3,4,5,6,7,8,9,10,11,12,13,14,21]
25	229.37	228.37	C_14_H_28_O_2_	myristic acid	fatty acids	[1,2,3,4,5,6,7,8,9,10,11,12,13,14,21]
26	257.43	256.42	C_16_H_32_O_2_	palmitic acid	fatty acids	[1,2,3,4,5,6,7,8,9,10,11,12,13,14,21]
27	271.49	270.50	C_17_H_34_O_2_	margaric acid	fatty acids	[1,2,3,4,5,6,7,8,9,10,11,12,13,14,21]
28	279.41	278.40	C_18_H_30_O_2_	linolenic acid	fatty acids	[1,2,3,4,5,6,7,8,9,10,11,12,13,14,21]
29	281.39	280.40	C_18_H_32_O_2_	linoleic acid	fatty acids	[1,2,3,4,5,6,7,8,9,10,11,12,13,14,21]
30	285.51	284.50	C_18_H_36_O_2_	stearic acid	fatty acids	[1,2,3,4,5,6,7,8,9,10,11,12,13,14,21]
31	297.39	296.40	C_18_H_32_O_3_	α-hydroxylinoleic acid	fatty acids	[1,2,3,4,5,6,7,8,9,10,11,12,13,14,21]
32	315.49	314.50	C_18_H_34_O_4_	octadecanedioic acid	fatty acids	[1,2,3,4,5,6,7,8,9,10,11,12,13,14,21]
33	321.49	320.50	C_20_H_32_O_3_	hydroxyarachidic acid	fatty acids	[1,2,3,4,5,6,7,8,9,10,11,12,13,14,21]
34	331.51	330.50	C_18_H_34_O_5_	trihydroxyoctadecenoic acid	fatty acids	[1,2,3,4,5,6,7,8,9,10,11,12,13,14,21]
35	341.59	340.60	C_22_H_44_O_2_	behemic acid	fatty acids	[1,2,3,4,5,6,7,8,9,10,11,12,13,14,21]
36	343.51	342.50	C_20_H_38_O_4_	eicosanedioic acid	fatty acids	[1,2,3,4,5,6,7,8,9,10,11,12,13,14,21]
37	355.59	354.60	C_23_H_46_O_2_	tricosanoic acid	fatty acids	[1,2,3,4,5,6,7,8,9,10,11,12,13,14,21]
38	369.61	368.60	C_24_H_48_O_2_	lignoceric acid	fatty acids	[1,2,3,4,5,6,7,8,9,10,11,12,13,14,21]
39	367.60	366.60	C_24_H_46_O_2_	nervonic acid	fatty acids	[1,2,3,4,5,6,7,8,9,10,11,12,13,14,21]
40	271.25	270.24	C_15_H_10_O_5_	apigenin	flavonoids	[1,2,3,4,5,6,7,8,9,10,11,12,13,14,21,64]
41	273.23	272.25	C_15_H_12_O_5_	naringenin	flavonoids	[1,2,3,4,5,6,7,8,9,10,11,12,13,14,21,64]
42	287.23	286.24	C_15_H_10_O_6_	kaempferol	flavonoids	[1,2,3,4,5,6,7,8,9,10,11,12,13,14,21,64]
43	291.27	290.27	C_15_H_14_O_6_	catechin	flavonoids	[1,2,3,4,5,6,7,8,9,10,11,12,13,14,21,64]
44	303.24	302.23	C_15_H_10_O_7_	quercetin	flavonoids	[1,2,3,4,5,6,7,8,9,10,11,12,13,14,21,64]
45	317.25	316.26	C_16_H_12_O_7_	isorhamnetin	flavonoids	[1,2,3,4,5,6,7,8,9,10,11,12,13,14,21,64]
46	319.23	318.23	C_15_H_10_O_8_	myricetin	flavonoids	[1,2,3,4,5,6,7,8,9,10,11,12,13,14,21,64]
47	373.41	372.40	C_20_H_20_O_7_	tangeretin	flavonoids	[1,2,3,4,5,6,7,8,9,10,11,12,13,14,21,64]
48	393.39	392.40	C_22_H_16_O_7_	luteolin	flavonoids	[1,2,3,4,5,6,7,8,9,10,11,12,13,14,21,64]
49	423.09	422.10	C_23_H_18_O_8_	interfungin B	flavonoids	[1,2,3,4,5,6,7,8,9,10,11,12,13,14,21,64]
50	429.51	428.50	C_26_H_28_N_4_O_2_	corin	flavonoids	[1,2,3,4,5,6,7,8,9,10,11,12,13,14,21,64]
51	451.39	450.40	C_21_H_22_O_11_	astilbin	flavonoids	[1,2,3,4,5,6,7,8,9,10,11,12,13,14,21,64]
52	477.41	476.40	C_26_H_20_O_9_	methylinoscavin A	flavonoids	[1,2,3,4,5,6,7,8,9,10,11,12,13,14,21,64]
53	565.49	564.50	C_26_H_28_O_14_	vicenin 1	flavonoids	[1,2,3,4,5,6,7,8,9,10,11,12,13,14,21,64]
54	579.51	578.50	C_27_H_30_O_14_	rhoifolin	flavonoids	[1,2,3,4,5,6,7,8,9,10,11,12,13,14,21,64]
55	581.49	580.50	C_27_H_32_O_14_	naringin	flavonoids	[1,2,3,4,5,6,7,8,9,10,11,12,13,14,21,64]
56	608.49	608.50	C_28_H_32_O_15_	diosmin	flavonoids	[1,2,3,4,5,6,7,8,9,10,11,12,13,14,21,64]
57	611.49	610.50	C_27_H_30_O_16_	rutin	flavonoids	[1,2,3,4,5,6,7,8,9,10,11,12,13,14,21,64]
58	139.13	138.12	C_7_H_6_O_3_	salicylic acid	phenolic acids	[1,2,3,4,5,6,7,8,9,10,11,12,13,14,21,64,72,73,74,75]
59	155.12	154.12	C_7_H_6_O_4_	protocatechuic acid	phenolic acids	[1,2,3,4,5,6,7,8,9,10,11,12,13,14,21,64,72,73,74,75]
60	165.15	164.16	C_9_H_8_O_3_	*p*-coumaric acid	phenolic acids	[1,2,3,4,5,6,7,8,9,10,11,12,13,14,21,64,72,73,74,75]
61	169.15	168.15	C_8_H_8_O_4_	vanillic acid	phenolic acids	[1,2,3,4,5,6,7,8,9,10,11,12,13,14,21,64,72,73,74,75]
62	171.11	170.12	C_7_H_6_O_5_	gallic acid	phenolic acids	[1,2,3,4,5,6,7,8,9,10,11,12,13,14,21,64,72,73,74,75]
63	179.19	178.18	C_10_H_10_O_3_	4-methoxy cinnamic acid	phenolic acids	[1,2,3,4,5,6,7,8,9,10,11,12,13,14,21,64,72,73,74,75]
64	181.15	180.16	C_9_H_8_O_4_	caffeic acid	phenolic acids	[1,2,3,4,5,6,7,8,9,10,11,12,13,14,21,64,72,73,74,75]
65	195.17	194.18	C_10_H_10_O_4_	ferulic acid	phenolic acids	[1,2,3,4,5,6,7,8,9,10,11,12,13,14,21,64,72,73,74,75]
66	199.17	198.17	C_9_H_10_O_5_	syringic acid	phenolic acids	[1,2,3,4,5,6,7,8,9,10,11,12,13,14,21,64,72,73,74,75]
67	355.31	354.31	C_16_H_18_O_9_	chlorogenic acid	phenolic acids	[1,2,3,4,5,6,7,8,9,10,11,12,13,14,21,64,72,73,74,75]
68	397.61	396.60	C_28_H_44_O	ergosterol	sterols	[1,2,3,4,5,6,7,8,9,10,11,12,13,14]
69	399.71	398.70	C_28_H_46_O	brassicasterol	sterols	[1,2,3,4,5,6,7,8,9,10,11,12,13,14]
70	415.71	414.70	C_29_H_50_O	β-sitosterol	sterols	[1,2,3,4,5,6,7,8,9,10,11,12,13,14]
71	427.69	426.70	C_30_H_50_O	lanosterol	sterols	[1,2,3,4,5,6,7,8,9,10,11,12,13,14]
72	179.17	178.18	C_10_H_10_O_3_	osmundacetone	terpenes	[1,2,3,4,5,6,7,8,9,10,11,12,13,14,64,72,74,75,76,77]
73	203.33	202.33	C_15_H_22_	α-curcumene	terpenes	[1,2,3,4,5,6,7,8,9,10,11,12,13,14,64,72,74,75,76,77]
74	205.34	204.35	C_15_H_24_	bergamotene	terpenes	[1,2,3,4,5,6,7,8,9,10,11,12,13,14,64,72,74,75,76,77]
75	219.33	218.33	C_15_H_22_O	α-turmerone	terpenes	[1,2,3,4,5,6,7,8,9,10,11,12,13,14,64,72,74,75,76,77]
76	223.36	222.37	C_15_H_26_O	β-eudesmol	terpenes	[1,2,3,4,5,6,7,8,9,10,11,12,13,14,64,72,74,75,76,77]
77	255.37	254.36	C_15_H_26_O_3_	inonotin I	terpenes	[1,2,3,4,5,6,7,8,9,10,11,12,13,14,64,72,74,75,76,77]
78	307.51	306.50	C_20_H_34_O_2_	fusicoserpenol A	terpenes	[1,2,3,4,5,6,7,8,9,10,11,12,13,14,64,72,74,75,76,77]
79	313.39	312.40	C_21_H_28_O_2_	inonotusic acid	terpenes	[1,2,3,4,5,6,7,8,9,10,11,12,13,14,64,72,74,75,76,77]
80	425.69	424.70	C_30_H_48_O	lupenone	terpenes	[1,2,3,4,5,6,7,8,9,10,11,12,13,14,64,72,74,75,76,77]
81	427.59	426.60	C_28_H_42_O_3_	9,11-dehydroergosterol peroxide	terpenes	[1,2,3,4,5,6,7,8,9,10,11,12,13,14,64,72,74,75,76,77]
82	427.71	426.70	C_30_H_50_O	lupeol	terpenes	[1,2,3,4,5,6,7,8,9,10,11,12,13,14,64,72,74,75,76,77]
83	429.61	428.60	C_28_H_44_O_3_	ergosterol peroxide	terpenes	[1,2,3,4,5,6,7,8,9,10,11,12,13,14,64,72,74,75,76,77]
84	441.71	440.70	C_30_H_48_O_2_	inoterpene F	terpenes	[1,2,3,4,5,6,7,8,9,10,11,12,13,14,64,72,74,75,76,77]
85	443.71	442.70	C_30_H_50_O_2_	betulin	terpenes	[1,2,3,4,5,6,7,8,9,10,11,12,13,14,64,72,74,75,76,77]
86	457.71	456.70	C_30_H_48_O_3_	trametenolic acid	terpenes	[1,2,3,4,5,6,7,8,9,10,11,12,13,14,64,72,74,75,76,77]
87	451.29	450.30	C_23_H_14_O_10_	inonoblin B	terpenes	[1,2,3,4,5,6,7,8,9,10,11,12,13,14,64,72,74,75,76,77]
88	457.69	456.70	C_30_H_48_O_3_	ganodecochlearin A	terpenes	[1,2,3,4,5,6,7,8,9,10,11,12,13,14,64,72,74,75,76,77]
89	459.37	458.37	C_30_H_50_O_3_	inonotus oxide B	terpenes	[1,2,3,4,5,6,7,8,9,10,11,12,13,14,64,72,74,75,76,77]
90	459.69	458.70	C_30_H_50_O_3_	inonotusane A	terpenes	[1,2,3,4,5,6,7,8,9,10,11,12,13,14,64,72,74,75,76,77]
91	459.73	458.72	C_30_H_50_O_3_	inonotus oxide A	terpenes	[1,2,3,4,5,6,7,8,9,10,11,12,13,14,64,72,74,75,76,77]
92	461.71	460.70	C_30_H_52_O_3_	inoterpene A	terpenes	[1,2,3,4,5,6,7,8,9,10,11,12,13,14,64,72,74,75,76,77]
93	469.71	468.70	C_31_H_48_O_3_	inonotusol F	terpenes	[1,2,3,4,5,6,7,8,9,10,11,12,13,14,64,72,74,75,76,77]
94	471.71	470.70	C_30_H_46_O_4_	inonotusolide B	terpenes	[1,2,3,4,5,6,7,8,9,10,11,12,13,14,64,72,74,75,76,77]
95	489.69	488.70	C_30_H_48_O_5_	inonotusol D	terpenes	[1,2,3,4,5,6,7,8,9,10,11,12,13,14,64,72,74,75,76,77]
96	585.41	584.40	C_36_H_56_O_6_	inonotustriol D triacetate	terpenes	[1,2,3,4,5,6,7,8,9,10,11,12,13,14,64,72,74,75,76,77]
97	247.21	246.21	C_13_H_10_O_5_	hispidin	styrylpyrones	[1,2,3,4,5,6,7,8,9,10,11,12,13,14,64,72,74,75,76,77]
98	393.39	392.40	C_22_H_16_O_7_	phelliribsin A	styrylpyrones	[1,2,3,4,5,6,7,8,9,10,11,12,13,14,64,72,74,75,76,77]
99	397.29	396.30	C_21_H_16_O_8_	inoscavin D	styrylpyrones	[1,2,3,4,5,6,7,8,9,10,11,12,13,14,64,72,74,75,76,77]
100	411.39	410.40	C_22_H_18_O_8_	methylinoscavin D	styrylpyrones	[1,2,3,4,5,6,7,8,9,10,11,12,13,14,64,72,74,75,76,77]
101	421.41	420.40	C_23_H_16_O_8_	inoscavin C	styrylpyrones	[1,2,3,4,5,6,7,8,9,10,11,12,13,14,64,72,74,75,76,77]
102	435.40	434.40	C_24_H_18_O_8_	methylinoscavin C	styrylpyrones	[1,2,3,4,5,6,7,8,9,10,11,12,13,14,64,72,74,75,76,77]
103	437.41	436.40	C_24_H_20_O_8_	inoscavin B	styrylpyrones	[1,2,3,4,5,6,7,8,9,10,11,12,13,14,64,72,74,75,76,77]
104	451.31	450.30	C_23_H_14_O_10_	inonoblin B	styrylpyrones	[1,2,3,4,5,6,7,8,9,10,11,12,13,14,64,72,74,75,76,77]
105	463.41	462.40	C_25_H_18_O_9_	inoscavin A	styrylpyrones	[1,2,3,4,5,6,7,8,9,10,11,12,13,14,64,72,74,75,76,77]
106	465.39	464.40	C_25_H_20_O_9_	davallialactone	styrylpyrones	[1,2,3,4,5,6,7,8,9,10,11,12,13,14,64,72,74,75,76,77]
107	475.39	474.40	C_25_H_14_O_10_	phelligridin E	styrylpyrones	[1,2,3,4,5,6,7,8,9,10,11,12,13,14,64,72,74,75,76,77]
108	477.41	476.40	C_26_H_20_O_9_	methylinoscavin A	styrylpyrones	[1,2,3,4,5,6,7,8,9,10,11,12,13,14,64,72,74,75,76,77]
109	479.41	478.40	C_26_H_22_O_9_	methyldavallialactone	styrylpyrones	[1,2,3,4,5,6,7,8,9,10,11,12,13,14,64,72,74,75,76,77]
110	479.45	478.45	C_26_H_22_O_9_	phelligridin F	styrylpyrones	[1,2,3,4,5,6,7,8,9,10,11,12,13,14,64,72,74,75,76,77]
111	595.51	594.50	C_32_H_18_O_12_	phelligridin G	styrylpyrones	[1,2,3,4,5,6,7,8,9,10,11,12,13,14,64,72,74,75,76,77]
112	179.21	178.20	C_10_H_10_O_3_	osmundacetone	other polyphenols	[1,2,3,4,5,6,7,8,9,10,11,12,13,14,64,72,74,75,76,77]
113	221.23	220.22	C_12_H_12_O_4_	hispolon	other polyphenols	[1,2,3,4,5,6,7,8,9,10,11,12,13,14,64,72,74,75,76,77]
114	229.24	228.24	C_14_H_12_O_3_	resveratrol	other polyphenols	[1,2,3,4,5,6,7,8,9,10,11,12,13,14,64,72,74,75,76,77]
115	303.19	302.19	C_14_H_6_O_8_	ellagic acid	other polyphenols	[1,2,3,4,5,6,7,8,9,10,11,12,13,14,64,72,74,75,76,77]
116	305.29	304.29	C_16_H_16_O_6_	inonophenol C	other polyphenols	[1,2,3,4,5,6,7,8,9,10,11,12,13,14,64,72,74,75,76,77]
117	317.23	316.22	C_15_H_8_O_8_	3-O-methylellagic acid	other polyphenols	[1,2,3,4,5,6,7,8,9,10,11,12,13,14,64,72,74,75,76,77]
118	465.39	464.40	C_25_H_20_O_9_	interfungin A	other polyphenols	[1,2,3,4,5,6,7,8,9,10,11,12,13,14,64,72,74,75,76,77]
119	151.13	150.13	C_5_H_10_O_5_	xylulose	carbohydrates	[1,2,3,4,5,6,7,8,9,10,11,12,13,14,64,72,74,75,76,77]
120	165.17	164.16	C_6_H_12_O_5_	rhamnose	carbohydrates	[1,2,3,4,5,6,7,8,9,10,11,12,13,14,64,72,74,75,76,77,78]
121	181.17	180.16	C_6_H_12_O_6_	inositol	carbohydrates	[1,2,3,4,5,6,7,8,9,10,11,12,13,14,64,72,74,75,76,77,78]
122	47.01	46.02	CH_2_O_2_	formic acid	organic acids	[1,2,3,4,5,6,7,8,9,10,11,12,13,14,64,72,74,75,76,77]
123	61.05	60.05	C_2_H_4_O_2_	acetic acid	organic acids	[1,2,3,4,5,6,7,8,9,10,11,12,13,14,64,72,74,75,76,77]
124	89.11	88.11	C_4_H_8_O_2_	butyric acid	organic acids	[1,2,3,4,5,6,7,8,9,10,11,12,13,14,64,72,74,75,76,77]
125	91.04	90.03	C_2_H_2_O_4_	oxalic acid	organic acids	[1,2,3,4,5,6,7,8,9,10,11,12,13,14,64,72,74,75,76,77]
126	199.14	198.13	C_8_H_6_O_6_	2,5-dihydroxylterephtalic acid	organic acids	[1,2,3,4,5,6,7,8,9,10,11,12,13,14,64,72,74,75,76,77]
127	227.45	226.44	C_16_H_34_	hexadecane	hydrocarbons	[1,2,3,4,5,6,7,8,9,10,11,12,13,14,64,72,74,75,76,77]
128	241.51	240.50	C_17_H_36_	heptadecane	hydrocarbons	[1,2,3,4,5,6,7,8,9,10,11,12,13,14,64,72,74,75,76,77]
129	255.49	254.50	C_18_H_38_	octadecane	hydrocarbons	[1,2,3,4,5,6,7,8,9,10,11,12,13,14,64,72,74,75,76,77]
130	297.61	296.60	C_21_H_44_	henicosane	hydrocarbons	[1,2,3,4,5,6,7,8,9,10,11,12,13,14,64,72,74,75,76,77]
131	109.15	108.14	C_7_H_8_O	benzyl alcohol	miscellaneous	[1,2,3,4,5,6,7,8,9,10,11,12,13,14,64,72,74,75,76,77]
132	111.12	110.11	C_6_H_6_O_2_	resorcinol	miscellaneous	[1,2,3,4,5,6,7,8,9,10,11,12,13,14,64,72,74,75,76,77]
133	139.13	138.12	C_7_H_6_O_3_	protocatechuic aldehyde	miscellaneous	[1,2,3,4,5,6,7,8,9,10,11,12,13,14,64,72,74,75,76,77]
134	161.23	160.22	C_10_H_12_N_2_	tryptamine	miscellaneous	[1,2,3,4,5,6,7,8,9,10,11,12,13,14,64,72,74,75,76,77]
135	175.25	174.24	C_11_H_14_N_2_	5-methyltryptamine	miscellaneous	[1,2,3,4,5,6,7,8,9,10,11,12,13,14,64,72,74,75,76,77]
136	287.50	286.50	C_20_H_30_O	retinol	miscellaneous	[1,2,3,4,5,6,7,8,9,10,11,12,13,14,64,72,74,75,76,77]
137	319.31	318.30	C_18_H_10_N_2_O_4_	melanin	miscellaneous	[1,2,3,4,5,6,7,8,9,10,11,12,13,14,64,72,74,75,76,77]
138	151.17	150.17	C_9_H_10_O_2_	benzyl acetate	ester	[1,2,3,4,5,6,7,8,9,10,11,12,13,14,64,72,74,75,76,77]
139	213.23	212.24	C_14_H_12_O_2_	benzyl benzoate	ester	[1,2,3,4,5,6,7,8,9,10,11,12,13,14,64,72,74,75,76,77]
140	229.25	228.25	C_14_H_12_O_3_	2-methoxyphenyl benzoate	ester	[1,2,3,4,5,6,7,8,9,10,11,12,13,14,64,72,74,75,76,77]
141	271.49	270.50	C_17_H_34_O_2_	methyl palmitate	ester	[1,2,3,4,5,6,7,8,9,10,11,12,13,14,64,72,74,75,76,77]
142	295.51	294.50	C_19_H_34_O_2_	methyl linoleate	ester	[1,2,3,4,5,6,7,8,9,10,11,12,13,14,64,72,74,75,76,77]

MS: Mass spectrometry.

**Table 3:** VOCs identified in the *I. obliquus* sample through MS.

VOC	Odor Profile
benzyl acetate	floral
α-curcumene	herbal
bergamotene	spice
β-eudesmol	woody
henicosane	waxy
benzyl alcohol	almond
resorcinol	phenolic
protocatechuic aldehyde	bitter
tryptamine	phenolic
formic acid	pungent
acetic acid	vinegar
retinol	floral
butyric acid	pungent
benzyl benzoate	balsamic
methyl linoleate	oily
2-methoxyphenyl benzoate	spicy
methyl palmitate	oily

MS: Mass spectrometry; VOC: Volatile organic compound.

**Table 4:** Particle diameter distribution of MIO and MIO–AgNP systems.

Sample	Particle Size Diameter (μm)		Volume Diameter (μm)		
	D[3,2]	D[4,3]	d_10_	d_50_	d_90_
MIO system	1.72 ± 0.011	2.45 ± 0.008	1.20 ± 0.016	2.10 ± 0.003	4.88 ± 0.019
MIO–AgNP system	1.65 ± 0.015	1.62 ± 0.002	0.25 ± 0.019	1.50 ± 0.005	3.05 ± 0.007

D[3,2] represents the surface-weighted mean diameter, and D[4,3] represents the volume-weighted mean diameter. The d_10_, d_50_, and d_90_ correspond to cumulative distributions at 10%, 50%, and 90%, respectively. MIO: Maltodextrin—*I. obliquus* MIO–AgNPs: Maltodextrin—*I. obliquus*-silver nanoparticles.

**Table 5:** Encapsulation parameters for the newly prepared *I. obliquus*-derived hybrid systems.

Sample	EE%	LC%	EY%
MIO system	77.65 ± 0.17	72.33 ± 0.11	74.58 ± 0.15
MIO–AgNP system	71.77 ± 0.07	68.55 ± 0.21	63.12 ± 0.14

EE%: Encapsulation efficiency; EY%: Encapsulation yield; LC%: Loading capacity; MIO: Maltodextrin—*I. obliquus* MIO–AgNPs: Maltodextrin—*I. obliquus*-silver nanoparticles.

**Table 6:** Evaluation of antibacterial performance against clinically relevant pathogenic strains.

Pathogenic Microorganism	Sample	Inhibition Zone Diameter (mm)						
		Sample Concentration (μg/mL)					Positive Control (Gentamicin, 100 μg/mL)	Negative Control (DMSO)
		100	125	150	175	200		
*Staphylococcus * *aureus*	*I. obliquus*	27.08 ± 0.17	38.15 ± 0.32	45.22 ± 0.19	50.38 ± 0.29	57.63 ± 0.29	22.21 ± 0.18	0
	citrate-coated AgNPs	13.01 ± 0.41	16.43 ± 0.42	19.02 ± 0.32	26.97 ± 0.55	30.14 ± 0.21		
	IO–AgNPs	39.05 ± 0.21	47.43 ± 0.33	60.18 ± 0.22	69.16 ± 0.13	74.55 ± 0.32		
	MIO system	29.72 ± 0.17	39.98 ± 0.44	47.21 ± 0.17	52.09 ± 0.14	59.06 ± 0.52		
	MIO–AgNP system	42.07 ± 0.32	49.75 ± 0.21	62.64 ± 0.33	73.05 ± 0.32	78.02 ± 0.44		
*Bacillus cereus*	*I. obliquus*	26.75 ± 0.31	33.24 ± 0.05	39.82 ± 0.16	42.13 ± 0.34	47.53 ± 0.27	18.24 ± 0.11	0
	citrate-coated AgNPs	38.06 ± 0.12	43.47 ± 0.16	47.02 ± 0.04	50.18 ± 0.21	53.07 ± 0.23		
	IO–AgNPs	43.75 ± 0.09	49.91 ± 0.17	52.38 ± 0.26	59.07 ± 0.31	63.58 ± 0.22		
	MIO system	29.89 ± 0.22	37.33 ± 0.07	43.53 ± 0.16	46.21 ± 0.24	51.19 ± 0.25		
	MIO–AgNP system	46.22 ± 0.19	52.21 ± 0.53	55.08 ± 0.15	62.75 ± 0.31	65.17 ± 0.32		
*Pseudomonas* *aeruginosa*	*I. obliquus*	16.54 ± 0.11	24.12 ± 0.21	39.76 ± 0.12	46.63 ± 0.16	55.32 ± 0.21	30.52 ± 0.23	0
	citrate-coated AgNPs	9.82 ± 0.14	11.63 ± 0.14	13.79 ± 0.23	16.42 ± 0.34	18.47 ± 0.27		
	IO–AgNPs	29.43 ± 0.18	47.65 ± 0.32	54.79 ± 0.43	60.56 ± 0.16	67.87 ± 0.23		
	MIO system	19.35 ± 0.22	28.05 ± 0.23	44.21 ± 0.17	50.64 ± 0.28	58.61 ± 0.32		
	MIO–AgNP system	34.58 ± 0.32	52.53 ± 0.23	58.04 ± 0.17	65.01 ± 0.26	71.19 ± 0.31		
*Escherichia coli*	*I. obliquus*	18.25 ± 0.31	25.42 ± 0.21	30.51 ± 0.19	39.44 ± 0.12	43.43 ± 0.27	20.53 ± 0.33	0
	citrate-coated AgNPs	13.11 ± 0.17	17.24 ± 0.23	20.08 ± 0.26	22.19 ± 0.32	25.83 ± 0.33		
	IO–AgNPs	28.03 ± 0.24	35.22 ± 0.46	42.54 ± 0.23	50.32 ± 0.18	54.29 ± 0.45		
	MIO system	20.43 ± 0.05	29.12 ± 0.31	33.43 ± 0.28	42.53 ± 0.37	47.92 ± 0.25		
	MIO–AgNP system	32.41 ± 0.15	41.43 ± 0.25	45.73 ± 0.31	50.21 ± 0.43	57.72 ± 0.43		

Values are expressed as the mean ± SD (*n* = 3). DMSO: Dimethyl sulfoxide; SD: Standard deviation.

**Table 7:** MIC and MBC values of the samples against representative pathogenic strains.

Pathogenic Microorganism	Sample	MIC (μg/mL)	MBC (μg/mL)	Gentamicin	
				MIC (μg/mL)	MBC (μg/mL)
*Staphylococcus aureus*	*I. obliquus*	0.25 ± 0.04	0.24 ± 0.12	0.62 ± 0.02	0.62 ± 0.02
	citrate-coated AgNPs	0.14 ± 0.07	0.13 ± 0.08		
	IO–AgNPs	0.12 ± 0.02	0.11 ± 0.06		
	MIO system	0.22 ± 0.01	0.22 ± 0.13		
	MIO–AgNP system	0.08 ± 0.05	0.09 ± 0.04		
*Bacillus cereus*	*I. obliquus*	1.09 ± 0.11	1.10 ± 0.17	1.30 ± 0.03	1.29 ± 0.02
	citrate-coated AgNPs	10.04 ± 0.24	10.02 ± 0.23		
	IO–AgNPs	0.81 ± 0.17	0.82 ± 0.33		
	MIO system	0.93 ± 0.08	4.07 ± 0.12		
	MIO–AgNP system	0.78 ± 0.09	0.95 ± 0.03		
*Pseudomonas aeruginosa*	*I. obliquus*	1.07 ± 0.08	1.06 ± 0.53	1.95 ± 0.22	1.96 ± 0.24
	citrate-coated AgNPs	0.68 ± 0.11	0.67 ± 0.21		
	IO–AgNPs	0.44 ± 0.12	0.45 ± 0.43		
	MIO system	0.97 ± 0.02	0.98 ± 0.09		
	MIO–AgNP system	0.38 ± 0.13	0.36 ± 0.16		
*Escherichia coli*	*I. obliquus*	0.47 ± 0.13	0.46 ± 0.08	1.12 ± 0.23	1.12 ± 0.22
	citrate-coated AgNPs	0.30 ± 0.11	0.31 ± 0.13		
	IO–AgNPs	0.28 ± 0.31	0.29 ± 0.07		
	MIO system	0.41 ± 0.04	0.42 ± 0.13		
	MIO–AgNP system	0.23 ± 0.21	0.25 ± 0.03		

Values are expressed as the mean ± SD (*n* = 3). MBC: Minimum bactericidal concentration; MIC: Minimum inhibitory concentration; SD: Standard deviation.

## Data Availability

The original contributions presented in this study are included in the article. Further inquiries can be directed to the corresponding author.

## Abbreviations

The following abbreviations are used in this manuscript:

AChE	Acetylcholinesterase
Ag	Silver
AgNPs	Silver nanoparticles
AMR	Antimicrobial resistance
ANOVA	Analysis of variance
ATCC	American Type Culture Collection
ATR	Attenuated total reflectance
Bcl-2	B-cell lymphoma 2
CFU	Colony-forming units
CO_2_	Carbon dioxide
CV	Coefficient of variation
D[3,2]	Surface-weighted mean diameter
D[4,3]	Volume-weighted mean diameter
d_50_	Median particle diameter
DLS	Dynamic light scattering
DMEM	Dulbecco’s Modified Eagle’s Medium
DMSO	Dimethyl sulfoxide
DNA	Deoxyribonucleic acid
DPPH	2,2-Diphenyl-1-picrylhydrazyl
DSC	Differential scanning calorimetry
DTA	Differential thermal analysis
DTG	Differential thermogravimetry
EDS	Energy-dispersive X-ray spectroscopy
EDX	Energy-dispersive X-ray
EE%	Encapsulation efficiency
ESI	Electrospray ionization
EY%	Encapsulation yield
FBS	Fetal bovine serum
FCC	Face-centered cubic
FeCl_3_	Ferric chloride
FEG	Field emission gun
FeSO_4_·7H_2_O	Ferrous sulfate heptahydrate
FRAP	Ferric reducing antioxidant power
FTIR	Fourier-transform infrared
GAE	Gallic acid equivalents
GC	Gas chromatography
HCl	Hydrochloric acid
HF	Heat flow
HSD	Honestly significant difference
IC_50_	Half-maximal inhibitory concentration
ICDD	International Centre for Diffraction Data
IO	*Inonotus obliquus*
IZ	Inhibition zone
JCPDS	Joint Committee on Powder Diffraction Standards
LC%	Loading capacity
MAPK	Mitogen-activated protein kinase
MBC	Minimum bactericidal concentration
MIC	Minimum inhibitory concentration
MIO	Maltodextrin—*I. obliquus*
MS	Mass spectrometry
MTT	3-(4,5-Dimethylthiazol-2-yl)-2,5-diphenyltetrazolium bromide
NIST	National Institute of Standards and Technology
NP	Nanoparticle
PDI	Polydispersity index
PI3K/Akt	Phosphoinositide 3-kinase/protein kinase B
PSD	Particle size distribution
QTOF	Quadrupole time-of-flight
RI	Retention index
ROS	Reactive oxygen species
SD	Standard deviation
SEM	Scanning electron microscopy
TPC	Total phenolic content
TPTZ	2,4,6-Tris(2-pyridyl)-1,3,5-triazine
t_R_	Retention time
TG	Thermogravimetry
TGA	Thermogravimetric analysis
UAE	Ultrasound-assisted extraction
VOC	Volatile organic compound
WPPF	Whole powder pattern fitting
XRD	X-ray diffraction
